# New tetrahydroisoquinolines bearing nitrophenyl group targeting HSP90 and RET enzymes: synthesis, characterization and biological evaluation

**DOI:** 10.1186/s13065-025-01399-0

**Published:** 2025-02-21

**Authors:** Etify A. Bakhite, Reda Hassanien, Nasser Farhan, Eman M. Sayed, Marwa Sharaky

**Affiliations:** 1 Chemistry Department, Faculty of Science, Assuit University, Assiut, 71516 Egypt; 2 Chemistry Department, Faculty of Science, New Valley University, El-Kharja, 72511 Egypt; 3 Pharmacology Unit, Cancer Biology Department, National Cancer Institute, Cairo University, 12613, El-Gize, Egypt

**Keywords:** Anticancer, Apoptosis, Cell cycle, RET enzyme (rearranged during transfection) enzyme, Heat shock protein (**HSP90**) enzyme, **HEPG2** cell line, **MCF7** cell line, Tetrahydroisoquinoline

## Abstract

In this study, new tetrahydroisoquinoline compounds were synthesized by reaction of 7-Acetyl-4-cyano-1,6-dimethyl-6-hydroxy-8- (3-nitrophenyl or 4-nitrophenyl)-5,6,7,8-tetrahydrosoquinoline-3(2*H*)-thiones with methyl iodide, chloro acetonitrile, ethyl chloroacetate to produce compounds **3–5** and reacted with *N*-arylchloroacetamides reagents to gave tetrahydroisoquinolin-3-ylthio) acetamides compounds **6a**–**c**, **8a**–**b** which can cyclized to 6,7,8,9-tetrahydrothieno[2,3-c]Isoquinoline-2-carboxamides compounds **7a**–**c, 9a**–**b**. Also react with *N*-(benzthiazol-2-yl)-2-chloroacetamideto give compound **10**. The structures of all newly synthesized compounds were characterized by elemental and spectral analyses. Also, most of the synthesized compounds were evaluated for their anticancer activities aganist **MCF7** and **HEPG2** cell lines**.** From the result we found that the most active compound against the **MCF7** cell lines was compound **8b,** and the most active compound against **HEPG2** cell lines was compound **3.** Then the effects of compound **3** on the **HEPG2** cell line was investigated using an apoptotic Annexin V-FITC test and flow cytometry. Compound **3** induced a 59-fold increase in **HEPG2** cell line apoptosis and cell cycle arrested at the G0-G1, G2/M phases. Moreover, the molecular docking study was applied and the result showed that compounds **8b** bind to the RET enzyme with binding energies of − **6.8** kcal/mol in comparison with standard **alectinib**, which exhibits a binding energy of − **7.2** kcal/mol. Compound **3** can bind with **HSP 90** with a binding energy (ΔG) of − 6.8 kcal/mol, which was comparable to the standard **Onalespib** (− 7.1 kcal/mol).

**Graphical Abstract:**

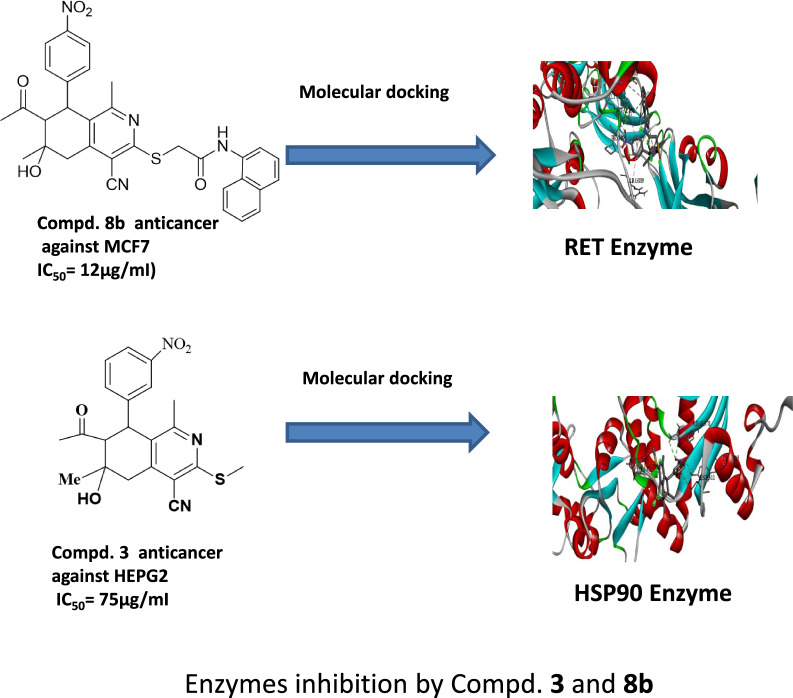

**Supplementary Information:**

The online version contains supplementary material available at 10.1186/s13065-025-01399-0.

## Introduction

Cancer is unbalanced cell proliferation results from changes in genetic expression, which causes cells to divide uncontrollably [1, 2]. Cancer is a form of hereditary disease. It is the main factor that causes mortality and morbidity [3]. Treating cancer diseases has been an important issue [4]. Common method, such as surgery, chemotherapy, and radiotherapy were used but in recent years more safe methods for cancer treatment are favorable like stem cell therapy, targeted therapy, ablation therapy, nanoparticles, natural antioxidants and new anticancer chemical compounds [4].

Liver cancer is the most prevalent cause of cancer-related fatalities, and it ranks sixth in the US [5]. Only this cancer has an annual percentage rise in frequency among the top five deadly cancers [6, 7]. Developed countries have Liver diseases are more common in developing nations [8]. Liver cancer can caused by hepatitis B and C viruses [9], fatty liver disease, cirrhosis caused by alcohol [7], smoking, obesity, diabetes, iron overload, and other dietary exposures are risk factors. Nevertheless, the success rate of chemotherapeutic therapy is less than one-third of patients, and after a few months of the program’s initiation, medication resistance develops [10, 11]. Eating more fruits and vegetables reduces the chance of acquiring cancer, according to a European study [10–12]. Some chemicals exhibited cytotoxicity to cancer cells while leaving non-cancerous cells unharmed [13] such as Piperine, inhibits enzymes required for drug metabolism, implying that co-administration with existing chemotherapeutic medicines may be used to raise plasma concentrations [14]. Furthermore, polysaccharides derived from Tricholoma matsutake and Lentinus edodes enhance the inhibitory effect of 5-fluorouracil (5-FU) [15]. Moreover, tetrahydroisoquinoline compounds have the potential to treat liver cancer. For example, 3-arylisoquinoline-based natural products based on corydamine exhibited a significant inhibitory effect, and mechanistic studies suggested that the compound was a dual inhibitor of Topo I and Topo II, with Topo II inhibitory activity [16]. Additionally, the 1-styrenyl isoquinoline compound demonstrated anticancer properties against Huh7 and SK-Hep-1 cells [17].

Breast cancer is the most frequent type of cancer in women worldwide, accounting for 2.26 million cases annually, 11.7% of all cancer cases, and 24.5% of female cancer cases [18, 19]. Cytotoxic chemotherapy medications, including capecitabine, 5-fluorouracil (5-FU), doxorubicin, epirubicin, gemcitabine, methotrexate, paclitaxel, tamoxifen citrate, and nucleosides, are the standard treatment for breast cancer [20]. They are still considered hazardous compounds even though they have been linked to negative long-term side effects [20, 21]. An ongoing research project is underway to develop effective new drugs or improve chemotherapy regimens.

A new compounds derivative based on tetrahydro-[1,2,4]triazolo[3,4-a]isoquinolin-3-yl)−3-arylprop-2-en-1-one was produced and evaluated on mouse (Luc-4T1) and human (MDA-MB-231) breast cancer cell lines [18]. Also, a cycloplatinated (II) complex based on isoquinoline alkaloid induces ferritinophagy-dependent ferroptosis in triple-negative breast cancer cells [22].

Another, methods for decrease cancer diseases spreading are inhibition the enzymes responsible for cancer multiplicity and spreading like DHFR, CDK2, RET, TULBIN, HSP90, HSP70, EGR…etc.

A molecular chaperone known as heat shock protein 90 (HSP90) is required for the stability and functionality of a number of conditionally activated and/or expressed signaling proteins [23, 24], as well as a number of mutant, chimeric, or overexpressed signaling proteins that promote the survival, proliferation, or both of cancer cells [25]. Through their specific engagement with a single molecular target, HSP90 inhibitors inactivate, disrupt, and eventually destroy HSP90 client proteins. It has shown promising antitumor potential in preclinical model systems [26–28].

The tyrosine kinase receptor, or RET, often engages in interactions with ligands at the cell surface and is essential for a variety of cellular processes, including as migration, metabolism, survival, differentiation, and proliferation [29]. At every stage of life, RET is expressed, starting at the very beginning of embryogenesis. A variety of aggressive diseases, such as Hirschsprung disease and cancer, are brought on by mutations that either activate or suppress RET [29]. Because the RET receptor is essential for hunger, weight gain management, and the survival and maintenance of multiple sclerosis, its important to inhibat RET enzyme to decrease disease spreading specially cancer diseases [30].

The 5,6,7,8-tetrahydroisoquinoline ring system is a structural element of numerous alkaloids [31, 32]. 5,6,7,8-Tetrahydroisoquinoline compounds have biological activities like enzyme inhibitors against many types of enzymes like DHFR, CDK2, RET, HSP90, EGFR and tulbin [33–36] and viral infections [37]. Also the were reported to have anticonvulsant properties [38]. Antibacterial [39], neurotropic [40], and antibacterial properties [41]. Also, 5,6,7,8-tetrahydroisoquinoline compounds have been used as anticancer agents [42–44].

On the other hand, numerous nitro-group-containing compounds have been shown to have a wide range of applications in biochemistry and medicine, including antioxidants and anticancers [45–48].

In light of the foregoing discoveries and as a continuation of our previous [49–52] work on tetrahyroisoquinolines, the purpose of this research was to synthesize and analyze the title compounds in the hope that these new compounds will find useful applications as anticancer drugs. And the difference between this work and pervious nitrophenyl tetrahydroisoquinoline work are: (a) In this article we synthesized meta and pare tetrahyderoisoquinolines but in the previous study we use ortho, tetrahyderoisoquinolines. (b) We used different cell lines HEPG2, MCF7. (c) We used different enzymes RET and HSP90.

## Results and discussion

### Synthesis part

**Scheme 1:**
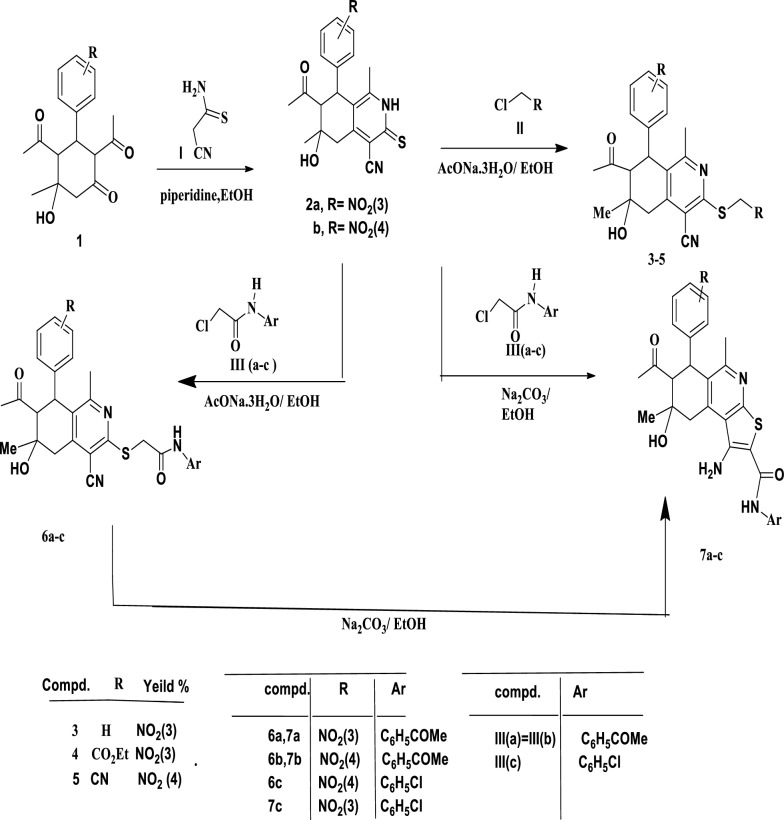
Synthesis of compounds **2a**–**b**, **3**–**5**, **6a**–**c**, **7a**–**c**

Cyclocondensation of 2,4-diacetyl-5-hydroxy-5-methyl-3-(2-nitrophenyl, 3-nitrophenyl, or 4-nitrophenyl)cyclohexanones **1a**–**b** with 2-cyanothioacetamide by refluxing in ethanol using pipridine as a basic catalyst formed the starting components are 7-acetyl-4-cyano-1,6-dimethyl-6-hydroxy-8-(3-nitrophenyl, or 4-nitrophenyl)−5,6,7,8-tetrahydrosoquinoline-3(2H)-thiones **2a**–**b** (Scheme 1).

Refluxing compounds **2a**–**b** with halocompounds like methyl iodide, ethyl chloroacetate, and chloroacetonitrile in ethanol with slightly excess sodium acetate trihydrate for one hour resulted in the formation of 3-(un)substituted methylthio-5,6,7,8-tetra-hydroisoquinoline-4-carbonitriles **3**–**5** (Scheme 1).

Also, compounds **2a**–**b** reacted with *N*-aryl-2-chloroacetamides **III(a**–**c**) to produce the corresponding *N*-aryl-(5,6,7,8-tetrahydroiso-quinolin-3-ylthio)acetamides **6a**–**c** in excellent yields. Compounds **6a**–**c** undergo cyclization by heating with catalytic quantities of sodium ethoxide in sodium carbonate 3 h to provide the equivalent 7-acetyl-1-amino-*N*-aryl-5,8-dimethyl-8-hydroxy-6-(3-nitrophenyl/4-nitrophenyl)−6,7,8,9-tetrahydrothieno[2,3-c]Isoquinoline-2-carboxamides **7a**–**c**. Compounds **7a**–**c** were also synthesized via heating compounds **2a**–**b** with the respective *N*-aryl-2-chloroacetamides **III(a**–**c**) in absolute ethanol in the presence of slightly excess molar amounts of sodium carbonate for 3 h (Scheme 1). Conversion of **6a**–**c** into the corresponding **7a**–**c** obeys intramolecular Thorpe-Ziegler cyclization.

**Scheme 2:**
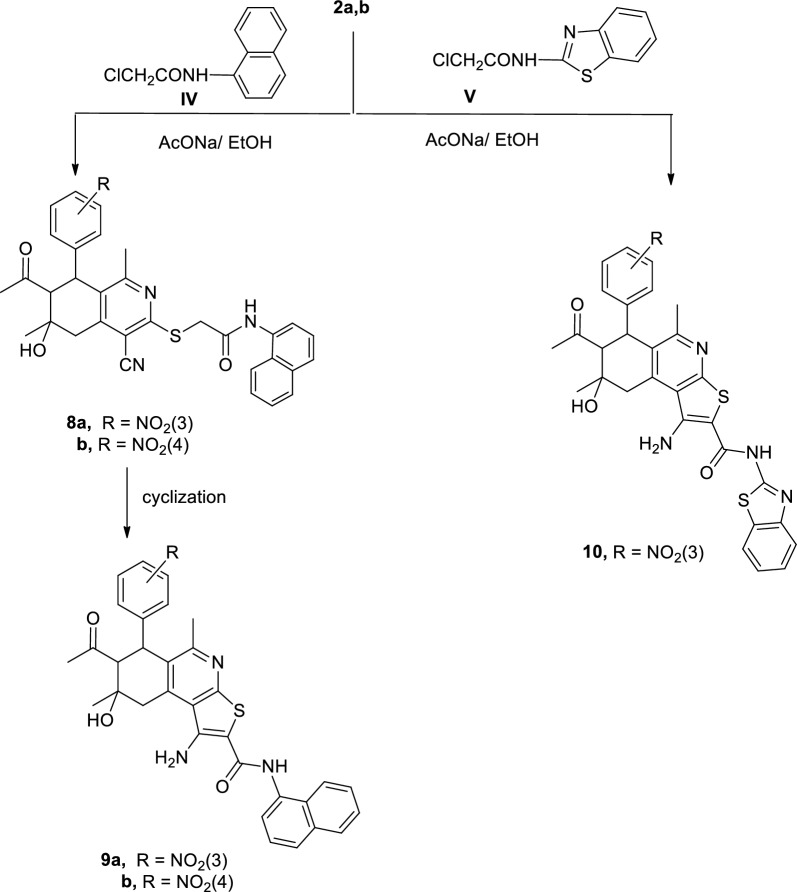
Synthesis of compounds **8a**, **b**, **9a**, **b** and **10**

In a similar manner, reaction of compound **2a**–**b** with *N*-(1-napthyl)−2-chloroacetamide (**IV**) by refluxing in ethanol, in the presence of slightly excess molar amounts of sodium acetate trihydrate, for one hour gave the corresponding *N*-(1-naphthyl)-(5,6,7,8-tetrahydroisoquinolin-3-ylthio)acetamides **8a**, **b** which can cyclized to **9a**, **b** (Scheme 2).

In contrast, reaction of **2a** with *N*-(benzthiazol-2-yl)−2-chloroacetamide **(V)** under the same (above) conditions yielded the cyclized form 1-amino-2-[(*N*-(benzthiazol-2-yl)]−6,7,8,9-tetrahydro-thieno[2,3-c]isoquinoline-2-carboxamide** 10** directly (Scheme 2).

The FT-IR, ^1^ HNMR, ^13^CNMR spectra of all synthesized compounds were in agreement with the expected results (supplementary date S1–S42).

### Cytotoxic activity

**Fig. 1:**
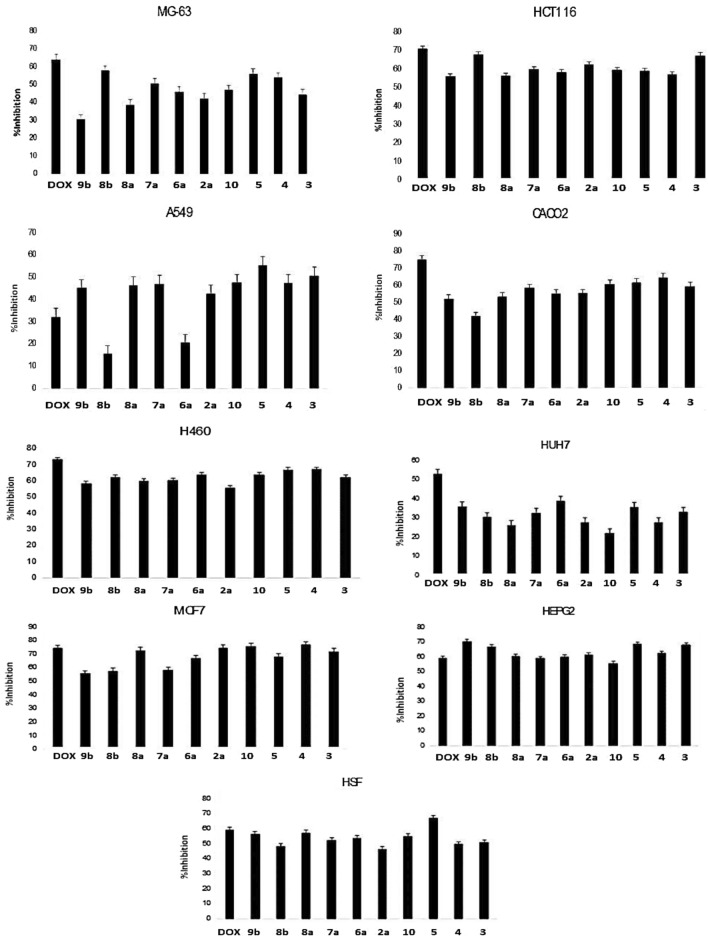
Cytotoxicity for one spot concentration 100 µg/ml of the synthesized compounds against nine cell lines

Compounds **3**–**5**, **12**, **2a**, **6a**, **7a**, **8a**, **8b** and **9b** were evaluated against nine human cancer cell lines at a single concentration point of 100 μg/ml to determine their inhibitory efficacy. The cell lines are: (human liver carcinoma **(HEPG2** and **HUH7**). human breast carcinoma (**MCF7)**, colon carcinoma human **(HCT116** and **CACO2)**. human lung carcinoma **(H460** and **A459).** human osteosarcoma **(MG-63)**. normal human skin cell line **(HSF)** (Table 1, Fig. 1). According to the results in (Table 1, Fig. 1), all synthetic compounds are more targeted and have a lower percentage of inhabitation against the human skin cell line HSF than doxorubicin (positive control), indicating that they are safer for normal cell lines. The raw data can be found in supplementary Table (S1–S4), Figure (S43–S44).

**Table 1:** Inhibition activity in one spot 100 µg/ml concentration of all compounds against normal skin cell line **HSF** in compared with Doxorubicin

Compd.no	Inhibition percent of **HSF** cell line	Compd.no	Inhibition percent of **HSF** cell line
**3**	52	**8a**	59
**4**	50	**8b**	50
**5**	70	**9b**	58
**10**	56	**Doxorubicin**	60
**2a**	48		
**6a**	56		
**7a**	55

Using the MTT test method, the in vitro cytotoxicity of our synthesized compounds at different doses ranging from 0 to 100 g/Ml was examined in two cell lines, **HEPG2** and **MCF7**, to examine 50% of the cancer cells dying.

**Fig. 2:**
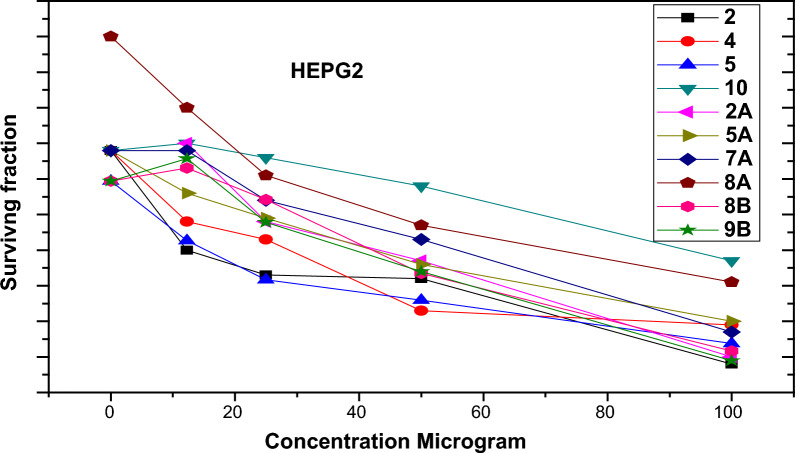
Surviving fraction of **HEPG2** cell lines after treatment by the synthesized compounds

According to the results (Fig. 2, Table 2) two compounds, **3** and **2a**, showed the most potent cytotoxic activity against **HEPG2**, with IC_50_ values of 75 and 82 g/ml, respectively when compared to the standard doxorubicin for the raw data, see (Table S3) in the accompanying information.

**Table 2:** IC_50_ of the synthesized compounds against **MCF7** and **HEPG2** cell lines and their selectivity index

Compd.no	IC_50_ FOR HSF ± S.D. μg/ml	IC_50_ for MCF7 ± S.D μg/ml	Selectivity Index = IC_50_ of HSF/ IC_50_ of MCF7	IC_50_ for HEPG2 ± S.D μg/ml	Selectivity Index = IC_50_ of HSF/IC_50_ of HEPG2
**3**	96	33 ± 0.034	2.9	75 ± 0.063	1.2
**4**	100	47 ± 0.055	2.1	80 ± 0.070	1.2
**5**	71	33 ± 0.042	2.1	97 ± 0.053	0.73
**10**	89	> 100	–	> 100	–
**2A**	100	35 ± 0.061	2.8	82 ± 0.054	1.2
**6A**	89	21 ± 0.057	4.2	> 100	–
**7A**	90	50 ± 0.031	1.8	97 ± 0.054	0.92
**8A**	84	50 ± 0.019	1.6	88 ± 0.072	0.95
**8B**	100	12 ± 0.056	8.3	94 ± 0.82	1.1
**9B**	86	80 ± 0.050	1.1	87 ± 0.61	0.98
**DOX**	83	4.58 ± 0.081	18.1	4.13 ± 0.054	20.1

**Fig. 3:**
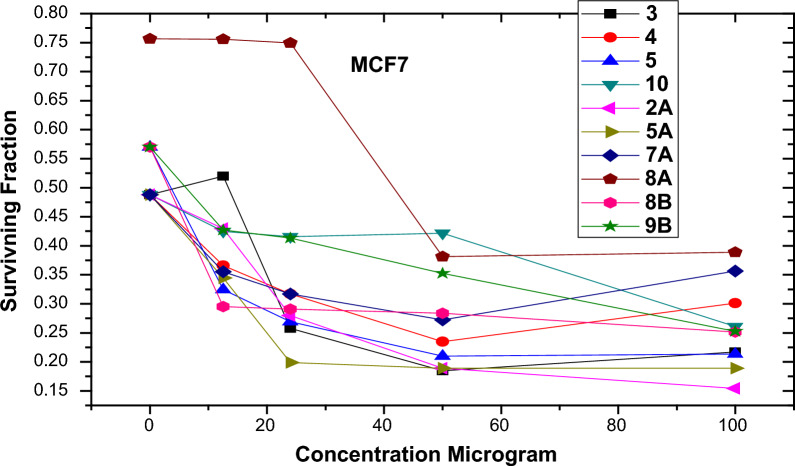
Surviving fraction of **MCF7** cell lines after treatment by the synthesized compounds

Also four compounds **8b**,** 3**,** 5** and **2a** exhibited significant cytotoxic action against **MCF7**, with corresponding IC_50_ values of **12**,** 33**,** 33** and **35** g/ml (Fig. 3, Table 2). For the raw data in the supporting information (Table S4).

BY calculating the selectivity index (SI) of the synthesized compounds and Doxorubicin. (SI) = IC_50_ of compound in normal cell lines \IC_50_ of the same compound in cancer cells [53, 54]. The synthesized compounds show selectivity index and the compounds **3**, **4**, **4**, **2a**, **6a** and **8b** showed **SI > 2** when divided on **MCF7** cell line that mean that this compounds safe and selective as anticancer drugs.

### Cell cycle arrest of HEPG2 Cells

**Fig. 4:**
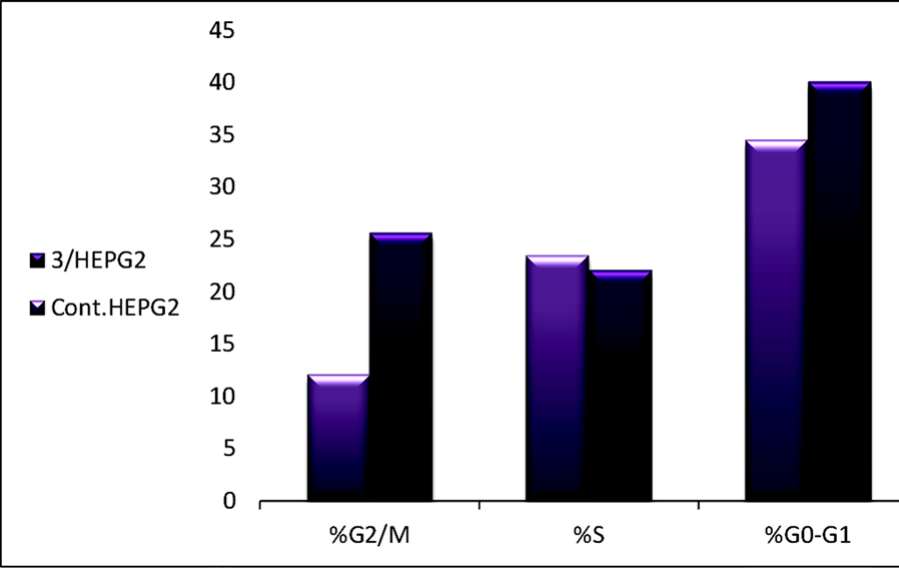
Cell cycle analysis of **HEPG2** treated with compound **3**

After adding compound **3**, we explore the growth inhibitory cell cycle mechanism of HEPG2 cell lines using DNA flow cytometry to examine the control and advancement of the cell cycle in HEPG2 cancer cells. Compound **3** was incubated with HEPG2 cells for 48 h at an IC_50_ of 75 µg/ml. When compound **3** was applied to HEPG2 cells, the cells cycled between the G0-G1 and G2/M phases (Fig. 4, Table 3). The G2/M phase fraction increased from 12.12% (in control cells) to 25.67%. Compound **3** can also stop HEPG2 cells at the G0–G1% stage of the cell cycle, with an increase in the G0–G1phase fraction from 34.54 to 40.10.

**Table 3:** Cell cycle analysis of **HEPG2** treated with compound **3**

Code	%G0–G1	%S	%G2/M
3/HEPG2	40.1	22.10	25.67
Cont.HEPG2	34.54	23.51	12.12

### Apoptosis induced

**Fig. 5:**
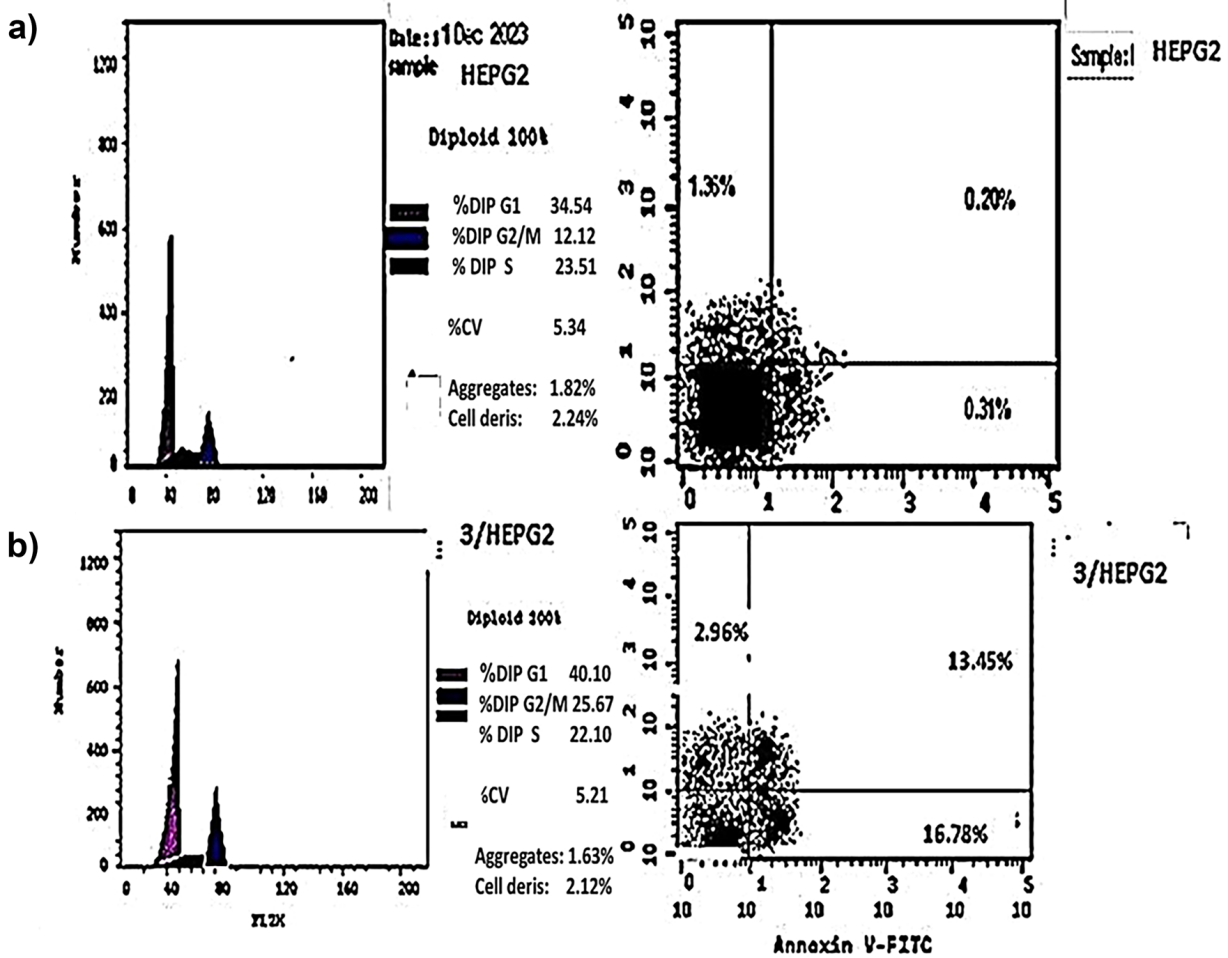
Apoptosis of **HEPG2** after treatment with compounds **3**. **a** Control HEPG2. **b** Compound **3/HEPG2**

**Fig. 6:**
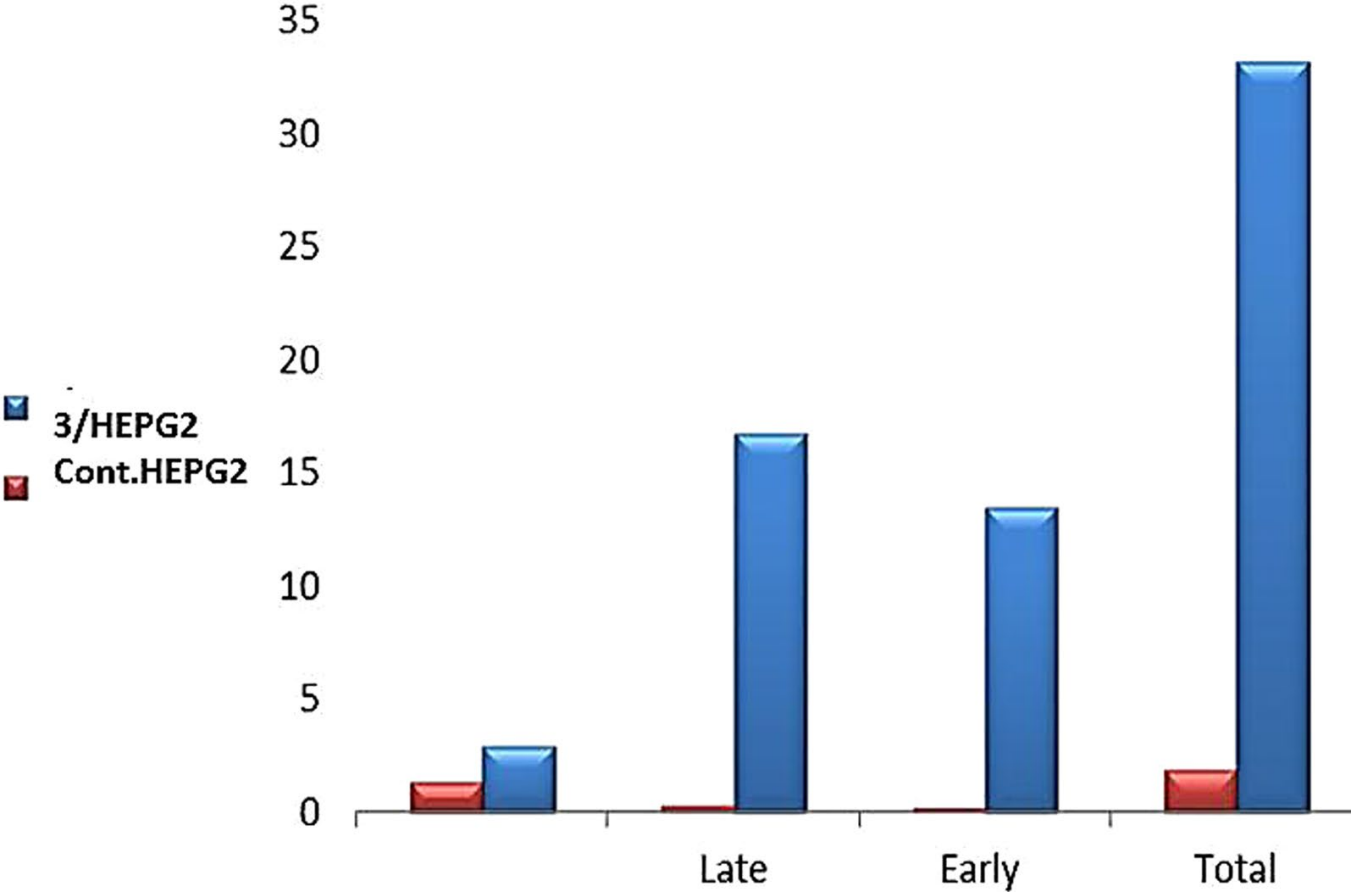
Apoptosis/necrosis assessment of **HEPG2** cells after treatment with compounds

When compound **3** was applied to HEPG2 cells, early and late cellular apoptosis increased (from 0.20 to 13.45%) and (from 0.31 to 16.78%), respectively, according to the results of the Annexin V-FITC/PI assay. Showing a marked rise in overall apoptosis relative to the untreated control. Furthermore, (Table 4 and Figs. 5 and 6) show that the percentage of necrotic cells increased from 1.36 to 2.96%. HEPG2 cell death increases by 59 times following treatment with compounds **3**. Therefore compound **3** has a biological mechanism that inhibits the proliferation of HEPG2 cells and has cytotoxic effects against cancer.

**Table 4:** Apoptosis/necrosis assessment of HEPG2 cells after treatment with compounds 3

Code	Apoptosis			Necrosis
	Total	Early	Late	
3/HEPG2	33.19	13.45	16.78	2.96
Cont.HEPG2	1.87	0.2	0.31	1.36

### Structure activity relationship (SAR)

**Scheme 3:**
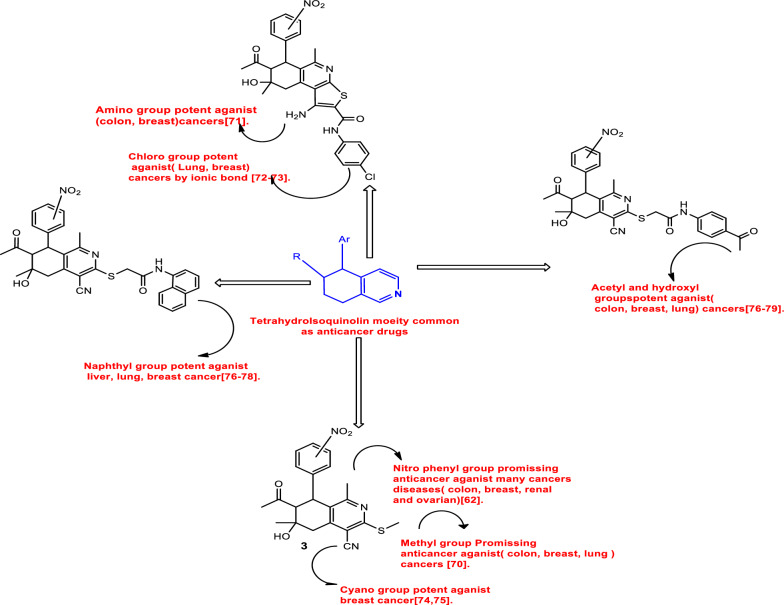
SAR study of interaction position of tetrahydroisoquinoline derivatives

The antiprofilate activity of tetrahydroisoquinoline is enhanced by its pyridine and benzene rings [31, 32, 43, 55–63]. Additionally, the three chiral centers in the synthesized compounds show the potential interaction with chiral biological molecules within cells, such as proteins, sugars, amino acids, enzymes, and nucleic acids [64–66]. The biomolecules can interact with the Asymmetric carbon centers with a variety of functional groups like acetyl, methyl, hydroxyl, cyano, amino in the cyclic form which found in our synthesized compounds. This interaction could result in biological activities such antioxidant, enzyme-inhibitory, and anticancer effects. Moreover, nitro groups allow for the formation of ionic connections with donor groups in the enzymes, which may enhance the biological activity [45–48]. Additionally, the synthesized compounds have high purity unless they three stereogenic centers, which means that each compound could have eight diastereomers. But due to the potential for hydrogen bonds between the acetyl and hydroxyl groups two chiral center cancel each other [49, 67–69]. According to the Structure–Activity Relationship (SAR) (Scheme 3) of the function group in the produced compounds and the anticancer activity, tetrahydroisoquinoline plays a significant role in biological activity [31, 32, 43, 55–60]. And the function groups were the mainly cause of biological activities. Compounds with carboxyl groups have been shown to exhibit anticancer properties against colon, breast, and lung cancer [70–72]. Methyl group-containing compounds are cytotoxic to lung, breast, and colon cancer cell lines [73]. Amino group-containing compounds show anticancer properties against lung and breast cancer [74]. Chlorine-containing compounds have anticancer effects on lung and breast cancer [75, 76]. Additionally, nitrile-group chemicals are cytotoxic to breast and lung cancer [77, 78]. Finally tetrahydroisoquinoline containing naphthyl groups are cytotoxic to liver, breast, and lung cancers [70, 79, 80].

### Molecular docking of the synthesized compounds binding with RET, HSP90 enzymes

**Fig. 7:**
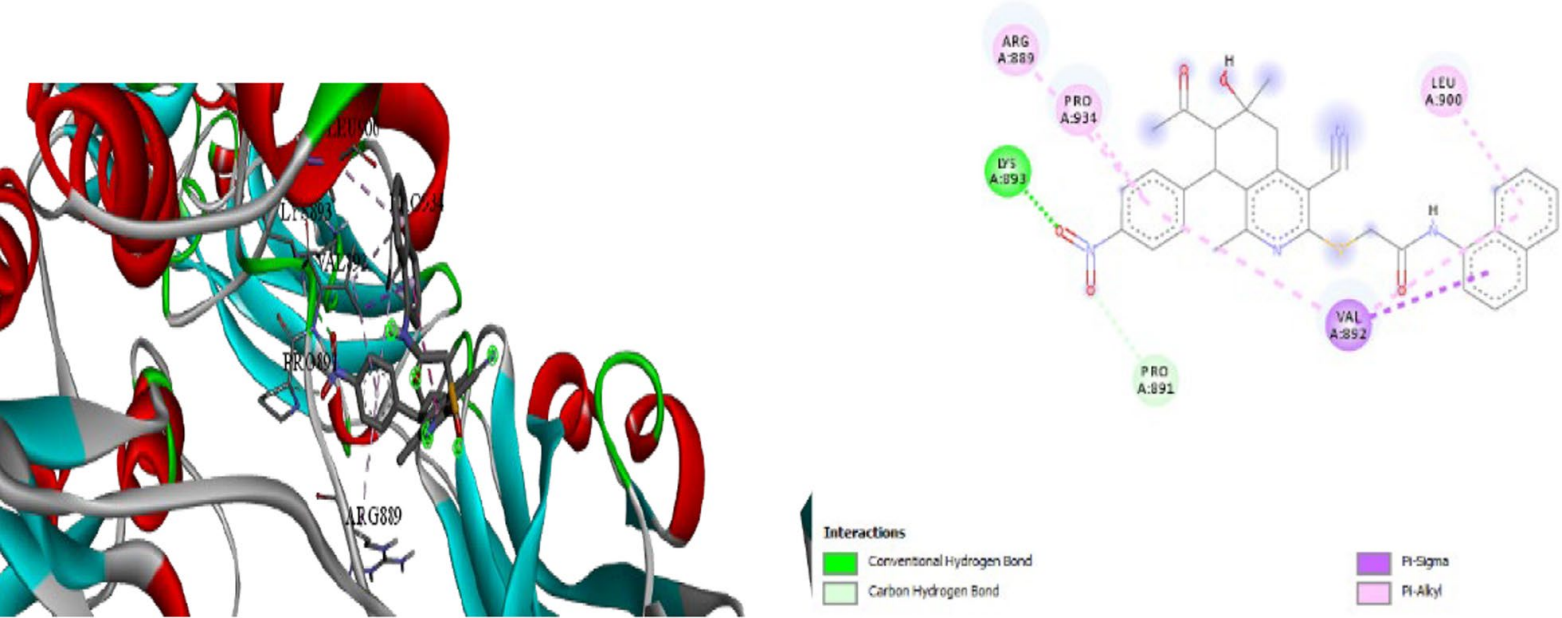
3D, 2D Molecular Docking of compound **8b** with **RET** receptor

**Fig. 8:**
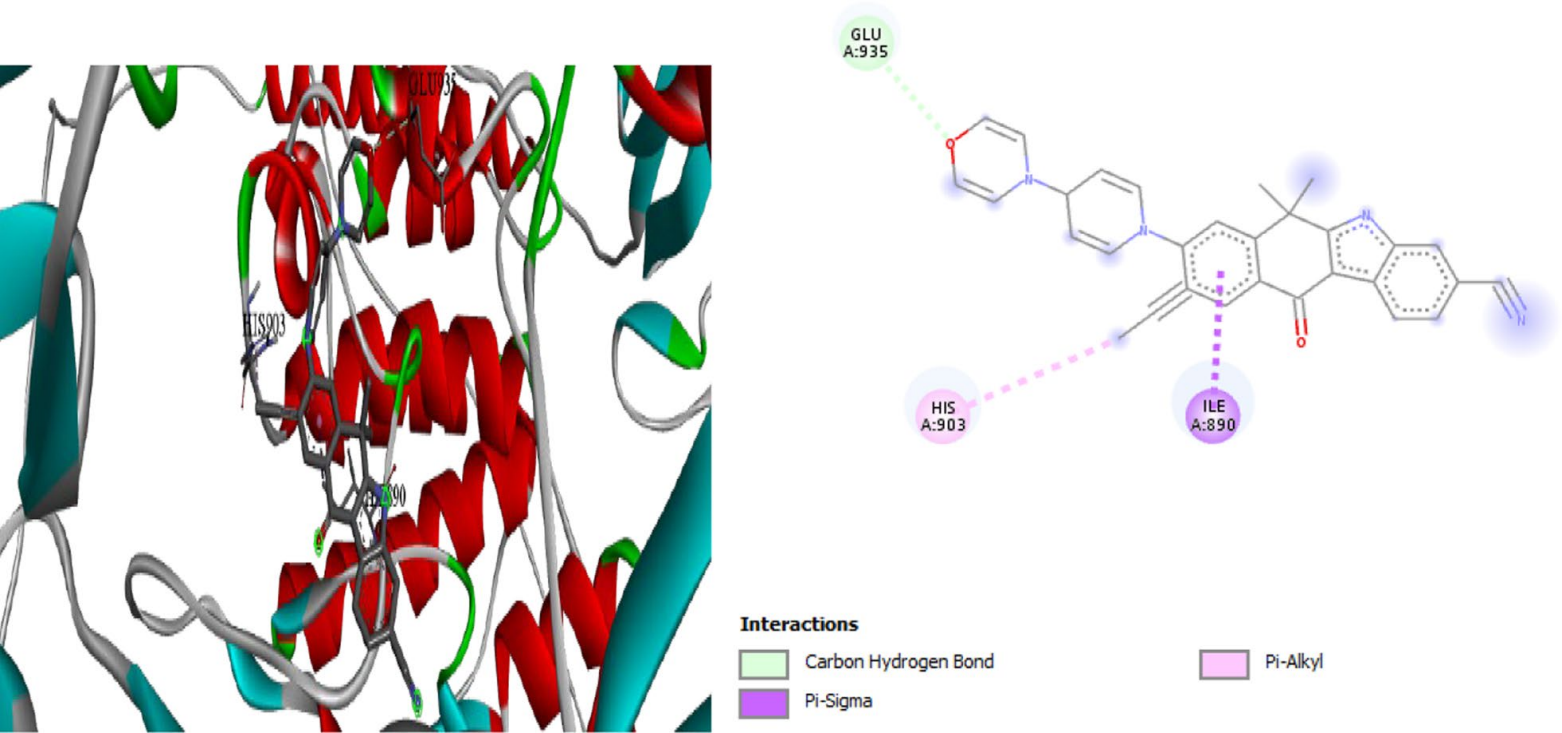
3D,2D Molecular Docking of standard (alectinib) with **RET** receptor

Molecular docking experiments were done in I Mole Lab for Bioinformatics in Cairo, Egypt. Table 5 displays the results of the molecular docking on the various ligand (compound) binding affinities with the **RET** enzyme (Rearranged during Transfection) was provided by the results. Because it participates in cell signaling pathways that are critical for cell growth, differentiation, and survival **RET** a tyrosine kinase receptor, is important for antiprolifate. Given that **RET** is linked to several cancers, including thyroid cancer and several forms of lung cancer, the biological activity of these compounds **8b** as potential **RET** inhibitors is very noteworthy. The ligand–protein interactions are numerically represented by the Gibbs free energy (∆G) for compound **8b** (− 6.8 kcal/mol). Values of the docking simulations; bigger negative values indicate higher binding. Compound **8b** demonstrated encouraging outcomes, forms a carbon hydrogen bond with, PRO892, Pi-sigma bond with VAL892, Pi-alkyl bond with ARG889, PRO931 and convential hydrogen bond with LYS893 (Figs. 7, 8, Table 5). In comparison with the standard compound (alectinib) (∆G) for compound **8b** (− 7.2 kcal/mol).exhibits interactions, primarily relying on a carbon-hydrogen bond and hydrophobic interactions (Fig. 8) for more details see the supplementary information (Table S5, S6).

**Table 5:** ∆G (kcal/mol) for each ligand with protein (RET)

Ligand	∆G (kcal/mol)
Compound 8b	− **6.8**
Standard (alectinib)	− **7.2**

The strong binding affinities observed, especially for compound **8b** suggest that these ligands may effectively inhibit RET kinase activity by occupying its ATP-binding site or inducing conformational changes that prevent kinase activation. This inhibition could potentially disrupt the aberrant signaling cascades associated with **RET** enzyme driven cancers.

In conclusion, this molecular docking study has identified several promising lead compounds, particularly compound **8b** which demonstrate strong binding affinities to the RET tyrosine kinase receptor. These findings provide a solid foundation for further optimization and development of potent RET inhibitors, potentially leading to new therapeutic options for RET-associated malignancies.

**Fig. 9:**
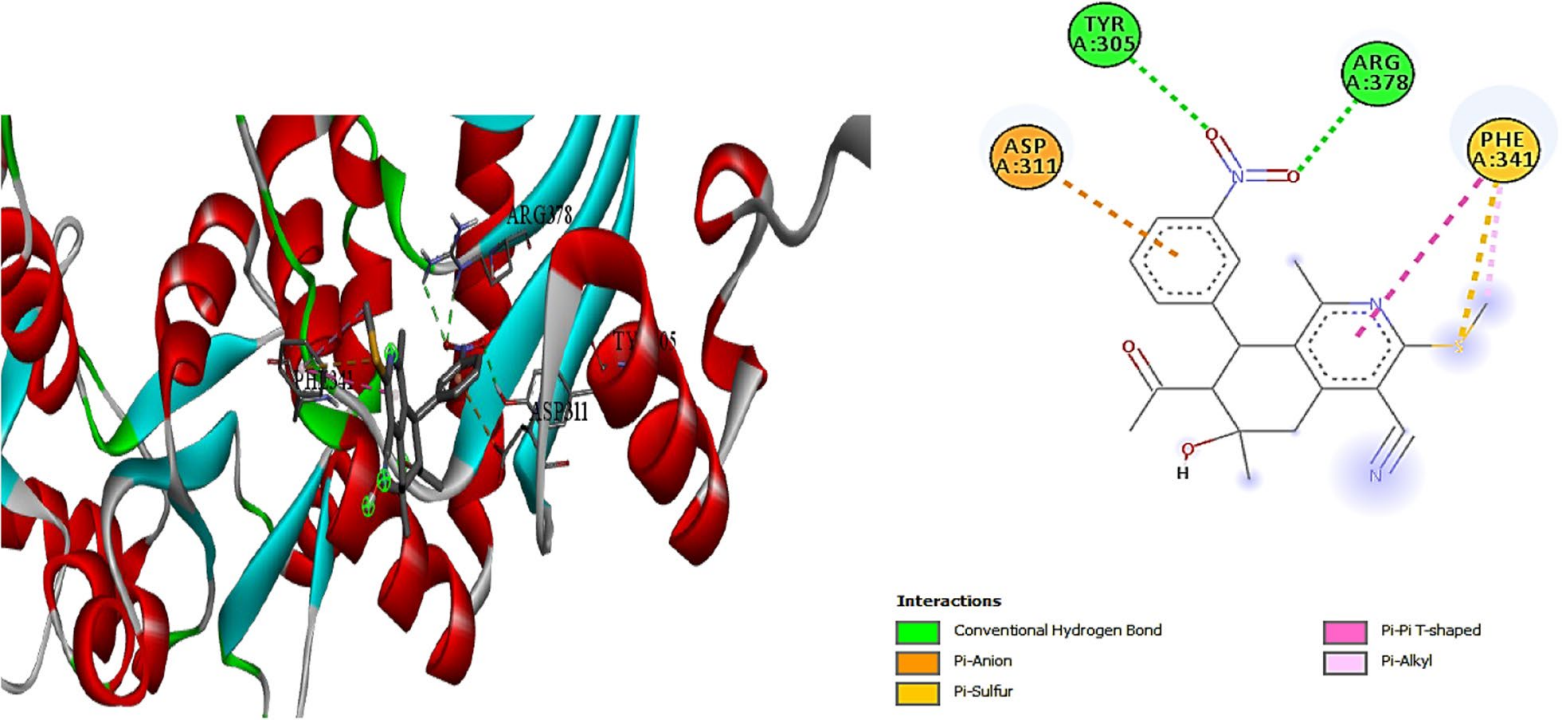
3D, 2D Molecular Docking of compound **3** with **HSP90** protein

**Fig. 10:**
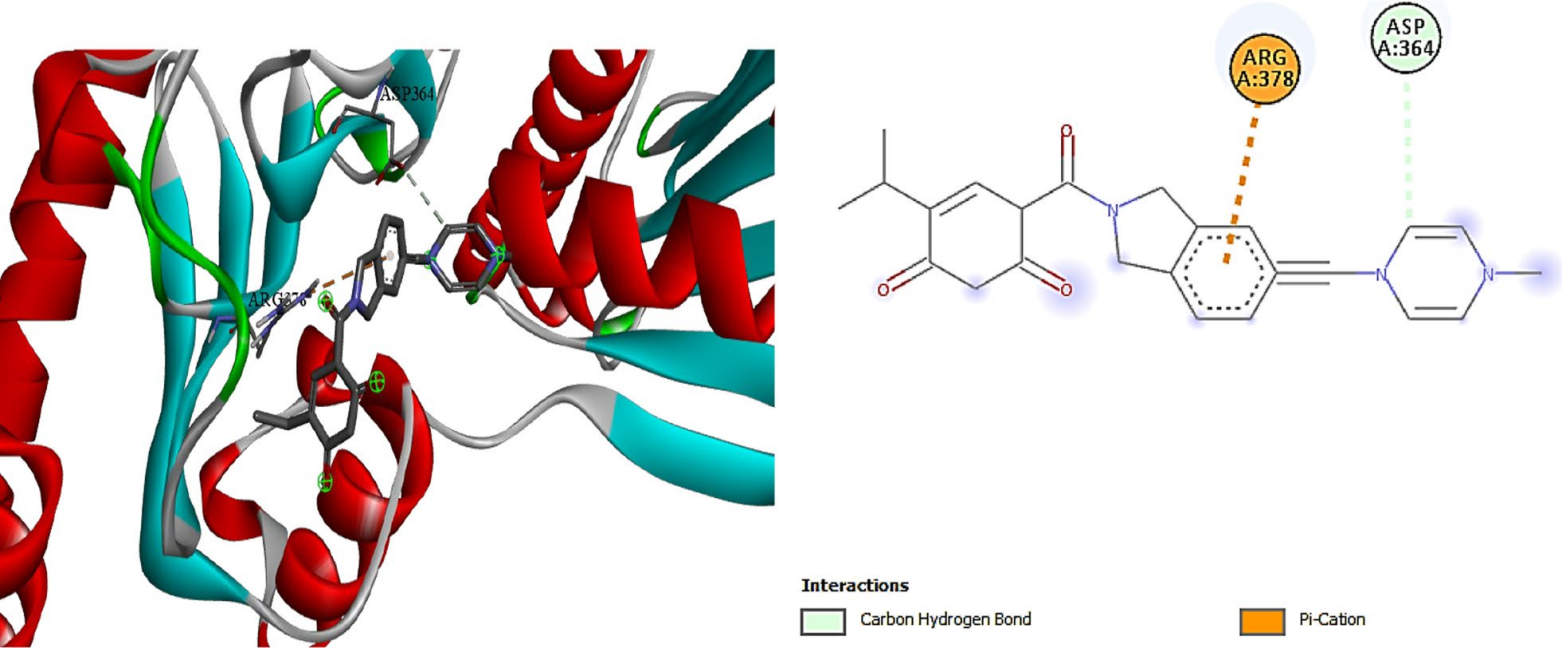
3D,2D Molecular Docking of Standard ligand (Onalespib)and protein **HSP90**

Also the molecular docking study for compound **3** was performed in (I Mole Lab for Bioinformatics-Cairo-Egypt) tested for binding with heat shock protein (HSP90) and the result showed that our tested compound **3** exhibited promising binding affinity to HSP90, with a binding energy (ΔG) of − 6.8 kcal/mol, which is comparable to the standard Onalespib (− 7.1 kcal/mol). This relatively small difference of 0.3 kcal/mol suggests that our tested compound could potentially serve as an effective HSP90 inhibitor, though slightly less potent than Onalespib (Table 6, Figs. 9, 10).

**Table 6:** ∆G (kcal/mol) and binding affinity for each compounds 3 tested with protein

compound	**∆G (kcal/mol)**
Standard (Onalespib)	− 7.1
Compound **3**	− 6.8

The detailed interaction analysis revealed that our tested compound **3** forms a complex network of interactions with HSP90, including three conventional hydrogen bonds with TYR305 and ARG378 residues, with distances ranging from 2.41 to 2.71 Å. These hydrogen bonds likely contribute significantly to the stability of the protein–ligand complex. The presence of a pi-anion interaction with ASP311 (4.04 Å) and a pi-sulfur interaction with PHE341 (5.27 Å) further strengthens the binding. Additionally, the compound forms hydrophobic interactions through pi-pi T-shaped and pi-alkyl interactions with PHE341, which are crucial for maintaining the proper orientation of the ligand within the binding pocket) for more details see the supplementary information (Tables S7, S8).

In contrast, the standered (Onalespib) showed a different interaction pattern, primarily forming a carbon hydrogen bond with ASP364 and a pi-cation interaction with ARG378. This distinct interaction profile, despite resulting in a slightly better binding energy, suggests that our tested compound **3** might offer unique advantages through its more diverse interaction network.

The biological implications of these findings are particularly noteworthy. HSP90 plays a crucial role in protein folding and stability, and its inhibition has been widely studied as a therapeutic strategy in various diseases, particularly cancer. The strong binding affinity and multiple interaction points observed with our tested compound **3** suggest it could effectively disrupt **HSP90** function. The involvement of TYR305 and ARG378 in hydrogen bonding is especially significant, as these residues are known to be important for **HSP90**’s chaperoning function.

From a toxicological perspective, the binding energy and interaction profile suggest that our compound **3** might have a favorable safety profile. The presence of multiple, specific interactions, rather than random binding, indicates selective targeting of **HSP90**. This specificity is crucial for minimizing off-target effects and potential toxicity. The similar binding energy to Onalespib, an established HSP90 inhibitor with known safety parameters, provides additional confidence in the potential safety profile of our tested compound.

These molecular docking results complete an important piece of the drug development puzzle by providing detailed structural insights into how our compound interacts with its target. The combination of favorable binding energy and specific molecular interactions suggests that our tested compound could be a viable candidate for further development as an HSP90 inhibitor. However, these computational findings should be validated through experimental studies to confirm the predicted binding mode and biological activity.

## Experimental

### Instrumentations

Instruments which used to measure the analysis of our synthesized compounds like melting point and ^1^HNMR, ^13^CNMR, FT-IR, Mass spectroscopy and elemental analysis were the same used in tour pervious work [50–52].

### Reaction of 2-acetylcyclohexanones 1a-b with cyanothioacetamide; synthesis of compounds 2a-b; general method

The general procedure for synthesis the starting material **2a**, **b** was mentioned before in our pervious published papers [50–52].

#### 7-Acetyl-4-cyano-1,6-dimethyl-6-hydroxy-8-(3-nitrophenyl)−5,6,7,8-tetra-hydroisoquinoline-3(2H)-thione (2a)

It is synthesized by reaction of **1a** with cyanothioacetamide Yield: 96%; m. p: 279–280 °C. Color: yellow to orange crystals. Recrystallized from ethanol. The FT-IR spectrum of compound **2a** show characteristics band at: 3429 cm^−1^ for (O–H), 3230 cm^−1^ for (N–H); 3139 cm^−1^ for (C–H, sp^2^); 2971 cm^−1^ for (C–H, sp^3^); 2221 cm^−1^ for (C≡N); 1710 cm^−1^ for (C=O). ^1^H NMR spectrum of compound **2a** (500 MHz, DMSO-*d*_*6*_) show signals at δ: 13.68 (s, 1H, NH); 7.95–8.05 (m, 2H, ArH); 7.51–7.58 (m, 2H, ArH); 5.05 (s, 1H, OH); 4.61–4.63 (d, *J* = 10 Hz, 1H, C^8^H); 3.23–3.26 (d, *J* = 15 Hz, 1H, C^5^H), 2.88–2.90 (d, *J* = 10 Hz, 1H, C^7^H), 2.83–2.87 (d, *J* = 20 Hz, 1H, C^5^H); 2.12 (s, 3H, COCH_3_); 1.84–1.86 (d, *J* = 10 Hz, 3H, CH_3_);1.23 (s, 3H, CH_3_). Anal. calcd for C_20_H_19_N_3_O_4_S (397.11): C, 60.44; H, 4.82; N, 10.57%. Found: C, 60.67; H, 5.11; N, 10.28%

#### 7-Acetyl-4-cyano-1,6-dimethyl-6-hydroxy-8-(4-nitrophenyl)−5,6,7,8-tetra-hydroisoquinoline-3(2H)-thione (2b)

It is synthesized by reaction of **1b** with cyanothioacetamide Yield: 93%; m. p 290–291 °C. The FT-IR spectrum of compound **2b** show characteristics band at: 3482 cm^−1^ for (O–H); 3235 cm^−1^ for (NH); 3106 cm^−1^ for (C–H, sp^2^); 2971, 2872 cm^−1^ for (C–H, sp^3^); 2220 cm^−1^ for (C≡N); 1708 cm^−1^ for (C=O). ^1^H NMR spectrum of compound **2b** in (500 MHz, DMSO-*d*_*6*_) show signals at δ: δ13.85(S, H, NH) 7.84–7.86 (d, *J* = 10 Hz, H, ArH); 7.62–7.64 (d, *J* = 10, H, ArH); 7.51–7.53 (d, *J* = 10 Hz, H, ArH); 7.33–7.34 (d, *J* = 5 Hz, H, Ar), 5.04 (s, 1H, OH); 4.97–4.99 (d, *J* = 10 Hz, 1H, C^8^H); 3.33 (s, 1H, C^5^H);, 3.16–3.10 (m, 1H, C^7^H), 2.86–2.90 (d, *J* = 20 Hz, 1H, C^5^H); 2.02 (s, 3H, COCH_3_); 1.93 (s, 3H, CH_3_); 1.29 (s, 3H, CH_3_). Anal. calcd for C_20_H_19_N_3_O_4_S (397.11): C, 60.44; H, 4.82; N, 10.57%. Found: C, 60.32; H, 5.04; N, 10.33%.

### Reaction of compounds 2a–b with methyl iodide, ethyl chloroacetate, chloroacetonitrile or its *N*-aryl-2-chloroacetamides III(a–c), IV; Synthesis of compounds 3–5, 6a–c and 8a,b general method (A) were carrying according to the general method in our perivous work [50–52]

A mixture of **2a**–**b** (10 mmol), a halocompound (10 mmol) was refluxed in ethanol in the presence of sodium acetate trihydrate (1.50 g, 11 mmol). The formed solid that were cooling and collected to recrystallized from ethanol to give crystals of compounds **3**–**5, 6a**–**c**.

#### 7-Acetyl-4-cyano-1,6-dimethyl-6-hydroxy-3-methylthio-8-(3-nitrophenyl)−5,6,7,8-tetrahydroisoquinoline (3)

It is synthesized by reaction of **2a** with methyliodide. Yield: 94%; 150 m.p.: 149–150 °C. The FT-IR spectrum of compound **3**: 3500 cm^−1^ for (O–H); 3077 cm^−1^ for (C–H, sp^2^); 2971–2931 cm^−1^ for (C–H, sp^3^); 2213 cm^−1^ for (C≡N); 1701 cm^−1^ for (C=O acetyl). ^1^H NMR spectrum of compound **3** in (400 MHz, DMSO-d_6_) show signals at δ: 7.95–8.09 (m, 2H, ArH); 7.55–7.59 (m, 2H, ArH); –4.98(s, 1H,OH), 4.77- 4.79 (d, J = 8 Hz, 1H, C^8^H),3.21–3.36 (m, 3H, CH_3_), 3.13–3.15 (t, J = 8 Hz, 1H, C^7^H); 2.87–2.95 (m,2 H, 2C^5^H); 2.18 (s, 3H, COCH_3_), 1.98 (s, 3H, CH_3_); 1.27–1.29 (s, 3H, CH_3_). ^13^C NMR δ 208.96, 160.66, 158.33, 150.10, 147.96, 146.09, 135.17, 130.18, 128.28, 122.69, 121.73, 115.18, 104.50, 67.40, 65.96,43.27, 42.9, 31.04, 27.54, 24.90, 23.79, 14.49. Anal. calcd for C_21_H_21_N_3_O_4_S (411.13): C, 61.30; H, 5.14; N, 10.21. found C, 62.18; H, 5.44; N, 10.00.

#### Ethyl 2-[(7-acetyl-4-cyano-1,6-dimethyl-6-hydroxy-8-(3-nitrophenyl)−5,6,7,8-tetra-hydroisoquinolin-3-yl)thio]acetate (4)

It is synthesized by reaction of **2a** with ethylchloroacetate Yield:93%; m.p.: 177–180 °C. The FT-IR spectrum of compound** 4**: 3495 cm^−1^ (O–H); 3079 cm^−1^ (C–H, sp^2^); 2984–2933 cm^−1^ (C–H, sp^3^); 2217 cm^−1^ (C≡N); 1723, 1700 cm^−1^ (C=O ester, acetyl). ^1^H NMR spectrum of compound **4** in (400 MHz, DMSO-d_6_) show signals at δ: 8.12–8.14 (m, 1H, ArH); 7.8 (s, 1H, ArH); 7.50–7.54 (t, J = 16 Hz, 1H, ArH); 7.35–7.36 (d, J = 4 Hz, 1H, ArH); 4.50–4.52 (d, J = 8 Hz 1H,OH), 4.11–4.14 (m, 2H, CH_2_ acetate), 3.91 (s, 6.8 Hz, 2H, C^8^ H_,_ C^5^H), 2.98–3.18 (m, 5.5 Hz, 4H, SCH_2_, C^7^H, C^5^H);1.85–1.88 (d, J = 12 Hz, 6H,CH_3_, COCH_3_), 1.38 (s, 3H,CH_3_);1.2 (s, 3H, CH_3_).

The mass spectrum show molecular ion peak at m/z [M+] = 483.11 in agreement with its molecular formula: (C_24_H_25_N_3_O_6_S) with the exact mass: 483.15. The most abundant peak (base peak) at 439 due to cleavage of ester group and form the aldehyde (C_22_H_22_N_3_O_5_S). Anal. calcd for C_24_H_27_N_3_O_6_S (485.16): C, 59.37; H, 5.60; N, 8.65 found C, 59.59; H, 5.20; N, 8.75.

#### 2-[(7-Acetyl-4-cyano-1,6-dimethyl-6-hydroxy-8-(4-nitrophenyl)−5,6,7,8-tetra-hydroisoquinolin-3-yl)thio]acetoonitriile (5)

It is synthesized by reaction of **2b** with chloroacetonitrile. Yield: 90%; m.p.:183–185 °C. The FT-IR spectrum of compound **5:** 3508 cm^−1^ for (O–H); 3108 cm^−1^ for (C–H, sp^2^); 2972, 2931 cm^−1^ for (C–H, sp^3^); 2250, 2216 cm^−1^ for (2C≡N); 1701 cm^−1^ for (C=O, acetyl). ^1^H NMR spectrum of compound **5** in (400 MHz, DMSO-d_6_) show signals at δ δ: 8.14–8.16 (d, J = 8 Hz, 2H, 2 Ar–H),7.37–7.39 (d, J = 8 Hz, 2H, 2Ar–H), 4.98 (s, 1H, OH), 4.80 (s, 1H, C^8^H), 4.28–4.33(m, 2H, SCH_2_), 3.32-3.36 (s, 1H, C^5^H), 2.92–2.99 (t, J = 12 Hz, 2H, C^7^H, C^5^H); 2.19 (d, 3H, COCH_3_), 2.05 (s, 3H, CH_3_);1.31 (s, 3H, CH_3_). ^13^C NMR spectrum of compound **5** (DMSO-d6) show environments of carbon as expected at δ: 209.08, 161.57, 155.28, 152.02, 151.10, 146.70, 130.07, 124.33, 118.02, 115.02, 106.8, 105, 67.95, 66.18, 56.52, 43.28, 40.66, 31.65, 27.97, 25.04, 18.98, 15.82. Anal. calcd for C_21_H_18_N_4_O_4_S (422.10): C, 59.70; H, 4.29; N, 13.26. Found C, 59.55; H, 4.30; N, 12.1%.

#### 2-[(7-Acetyl-4-cyano-1,6-dimethyl-6-hydroxy-8-(3-nitrophenyl)−5,6,7,8-tetra-hydroisoquinolin-3-yl)thio]-N-(4-acetylphenyl)acetamide (6a)

It was synthesized by reaction of **2a** with *N*-(4-acetylphenyl)−2-chloroacetamide. Yield: 93%; m.p.:231–232 °C. The FT-IR spectrum of compound **6a**: 3420 cm^−1^ (O–H); 3344 cm^−1^ (N–H); 2970 cm^−1^ (C–H, sp^2^); 2925 cm^−1^ (C–H, sp^3^); 2217 cm^−1^ (C≡N); 1702, 1674 cm^−1^ (C=Oamide, C=O acetyl). ^1^H NMR spectrum of compound **6a** in (400 MHz, DMSO-d_6_) show signals at δ: 10.56 (s, 1H, NH), 7.50–8.05 (m, 8H, 8ArH), 4.97 (s, 1H, OH), 4.73–4.75 (d, J = 8 Hz, 1H, C^8^H), 4.10–4.18 (m, 2H, SCH_2_), 3.31 (s, 1H, C^5^H) 2.87–2.95 (t, J = 8 Hz, 2H, C^7^H and C^5^H), 2.49 (s, 3H,COCH_3_), 2.15 (s, 3H, COCH_3_), 1.85 (s, 3H, CH_3_ attached to pyridine ring), 1.26 (s, 3H, CH_3_). ^13^C NMR spectrum of compound **6a** (DMSO-d6) show 27 environments of carbon as expected at δ 208.87, 196.18, 166.72, 160.49, 157.53, 150.23, 147.90, 145.96, 143.20, 135.12, 131.71, 130.15, 129.43, 128.70, 122.68, 121.70, 118.19, 115.02, 104.02, 67.53, 66.00, 43.26, 42.40, 34.85, 30.96, 27.47, 26.32, 24.57. Anal. Calcd. for C_30_H_28_N_4_O_6_S (572.17): C, 62.92; H, 4.93; N, 9.78%. Found: C, 62.95; H, 5.09; N, 9.75%.

#### 2-[(7-Acetyl-4-cyano-1,6-dimethyl-6-hydroxy-8-(4-nitrophenyl)−5,6,7,8-tetra-hydroisoquinolin-3-yl)thio]-N-(4-acetylphenyl)acetamide (6b)

It was synthesized by reaction of **2b** with *N*-(4-acetylphenyl)−2-chloroacetamide. Yield: 86%. M.p. 193–194 °C. The FT-IR spectrum of compound **6b**: 3540 cm^−1^ for (O–H); 3337 cm^−1^ for (N–H); 3109 cm^−1^ for (C–H, sp^2^); 2968 cm^−1^ for (C–H, sp^3^); 2220 cm^−1^ for (C≡N); 1683 cm^−1^ for (C=Oacetyl and amide), 1595 for (C=N). ^1^H NMR spectrum of compound **6b** in (400 MHz, DMSO-d_6_) show signals at δ: 10.57 (s, 1H, NH), 8.06–8.11 (d, J = 25 Hz, 2H, ArH), 7.84 (d, J = 10 Hz, 2H, ArH), 7.62– (s, ArH), 7.28–7.31 (d, J = 15 Hz, 2H, ArH), 5.02 (s, 1H, OH), 4.67–4.78 (s, 1H, C^8^H), 4.34 (s, 1H, C^5^H), 4.11 (s, 2H, SCH_2_), 2.88–2.89 (d, J = 5 Hz, 2H: C^7^H and C^5^H), 2.12 (s, 3H, COCH_3_), 1.80 (s, 3H, COCH_3_), 1.23 (s, 3H, CH_3_ attached to pyridine ring), 1.03 (s, 3H, CH_3_). Anal. Calcd for C_30_H_28_N_4_O_6_S (572.17): C, 62.92; H, 4.93; N, 9.78. Found: C, 63.00; H, 4.85; N, 10.06.

#### 2-[(7-Acetyl-4-cyano-1,6-dimethyl-6-hydroxy-8-(4-nitrophenyl)−5,6,7,8-tetra-hydroisoquinolin-3-yl)thio]-N-(4-chlorophenyl)acetamide (6c)

It was synthesized by reaction of **2b** with *N*-(4-chlorophenyl)−2-chloroacetamide Yield: 94%; m.p.: 144–145 °C. The FT-IR spectrum of compound **6c**: 3563 cm^−1^ for (O–H), 3344 cm^−1^ for (N–H); 3203 cm^−1^ for (C–H, sp^2^); 2972, 2937 cm^−1^ for (C–H, sp^3^); 2221 cm^−1^ for (C≡N); 1705 cm^−1^ for (C=O, acetyl); 1681 cm^−1^ for (C=O, amide). ^1^H NMR spectrum of compound **6c** in (400 MHz, DMSO-d_6_) show signals at δ: 10.35 (s, 1H, NH), 8.08–8.11 (m, 2H, ArH), 7.60–7.62 (d, J = 8 Hz, 2H, ArH), 7.29–7.54 (m, 4H, ArH), 4.98 (s, 1H, OH), 4.71–4.73 (d, J = 8 Hz, 1H, CH at C^8^), 4.06–4.14 (dd, J = 12,12 Hz, 2H, SCH_2_), 3.42–3.44 (d, J = 8 Hz, 1H, C^5^H), 2.90–2.92 (t, J = 10 Hz, 2H: C^7^H and C^5^H), 2.15(s, 3H, COCH_3_), 1.85 (s, 3H, CH_3_), 1.27 (s, 3H, CH_3_). ^13^C NMR spectrum of compound **6c** (DMSO-d6) show 28 environments of carbon as expected at δ: 208.53, 166.23, 164.75, 160.47, 157.63, 151.75, 150.04, 146.07, 137.85, 129.52, 128.73, 128.60, 126.83, 123.77, 120.90, 120.53, 114.98, 103.98, 67.39, 65.71, 55.99, 43.21, 42.65, 34.72, 31.02, 27.46, 24.45, 18.50. Anal. Calcd. for C_28_H_25_ClN_4_O_5_S (564.12): C, 59.52; H, 4.46; N, 9.92%. Found: C, 59.20; H, 4.67; N, 10.07%.

### Synthesis of 7-Acetyl-1-amino-2-(N-arylcarbamoyl)−5,8-dimethyl-8-hydroxy-6-(3-nitrophenyl or 4-nitrophenyl)−6,7,8,9-tetrahydrothieno[2,3-c]isoquinolines compounds 7a-c, 9a,b and 10 general method (B).

To a suspension of **6a**–**c**, **8a**, **b** (10 mmol) were refluxed in absolute ethanol (60 mL) using sodium carbonate for 3 h. The yellow solid that formed and recrystallized from ethanol to give **7a**–**c** according to the procedure in the pervious work [50–52].

#### 7-Acetyl-N-(4-acetylophenyl)−1-amino-5,8-dimethyl-8-hydroxy-6-(3-nitro-phenyl)−6,7,8,9-tetrahydrothieno[2,3-c]isoquinoline-2-carboxamide (7a)

It was obtained by cyclization of compound **6a** Yield: 90%; m.p.:277–280 °C. The FT-IR spectrum of compound **7a**: 3416, 3316 cm^−1^ for (O–H, NH_2_, NH); 2967, 2917 cm^−1^ for (C–H, sp^3^); 1701 cm^−1^ for (C=O). ^1^H NMR spectrum of compound **7a** in (400 MHz, DMSO-d_6_) show signals at δ: 9.73 (s, 1H, NH), 8.30 (s, 1H, Ar–H), 7.84–8.08 (m, 6H, 6Ar-H), 7.52–7.58 (m, 2H, NH_2_), 7.20 (s, 1H, Ar–H),4.86–4.88(d, J = 8 Hz, 2H, OH, C^6^H), 3.65–3.68 (s, 1H, C^9^H), 3.39–3.42 (d, J = 12 Hz, 1H, C^7^H), 2.93–2.95 (d, J = 8 Hz, 1H, C^9^H), 2.55 (s, 3H COCH_3_), 2.20 (s, 3H,CO CH_3_), 2.03 (s, 3H, CH_3_), 1.33 (s, 3H, CH_3_). ^13^C NMR spectrum of compound **7a** (DMSO-d6) show 25 environments of carbon as expected at δ: 209.43, 196.56, 164.35, 158.59, 156.80, 150.17, 147.93, 147.01, 143.60, 143.07, 135.50,131.12, 130.12,129.06, 125.35, 122.81, 122.43, 121.54, 119.93, 96.44, 79.14, 44.28, 42.89, 42.03, 31.17, 28.95, 27.93, 26.44, 24.77, 24.47, 22.08.

Anal. Calcd. for C_30_H_28_N_4_O_6_S (572.17): C, 62.92; H, 4.93; N, 9.78%. Found: C, 62.87; H, 5.29; N, 9.88%.

#### 7-Acetyl-N-(4-acetylophenyl)−1-amino-5,8-dimethyl-8-hydroxy-6-(4-nitro-phenyl)−6,7,8,9-tetrahydrothieno[2,3-c]isoquinoline-2-carboxamide (7b)

It was obtained by cyclization of compound **6b**. Yield: 89%; m.p.: 301–302 °C. IR; The FT-IR spectrum of compound **7b**: 3422, 3322 cm^−1^ for (O–H, NH_2_, NH); 2918 cm^−1^ for (C–H, sp^3^); 1702, 1679 cm^−1^ for (2 acetyl C=O). ^1^H NMR spectrum of compound **7b** in (400 MHz, DMSO-d_6_) show signals at δ: 9.71 (s, 1H, NH), 8.11–8.14 (t, j = 12 Hz, 3H, 3 Ar–H), 7.83 – 7.91 (m, 4H, 4Ar-H), 7.16–7.20 (m, 3H, Ar–H, NH_2_), 4.82–4.84(d, J = 8 Hz, 1H, OH), 3.59–3.70 (s, 1H, C^9^H), 3.40–3.52 (d, J = 12 Hz, 2H, C^6^H, C^7^H), 2.86–2.89 (d, J = 8 Hz, 1H, C^9^H), 2.52–2.58 (s, 3H COCH_3_), 2.45 (s, 3H,CO CH_3_), 1.99–2.00 (s, 3H, CH_3_), 1.30 (s, 3H, CH_3_). ^13^C NMR spectrum of compound **7b** (DMSO-d6) show 21 environments of carbon as expected at δ: ^13^C NMR δ:209.17, 169.80, 156.77, 153.03, 146.12, 132.10, 129.35, 129.02, 125.67, 123.78, 121.39, 120.13, 67.12, 66.11, 65.74, 43.17, 41.95, 31.17, 27.92, 26.35, 24.60. Anal. Calcd. for C_30_H_28_N_4_O_6_S (572.17): C, 62.92; H, 4.93; N, 9.78%. Found: C, 62.87; H, 5.29; N, 9.88%.

Anal. Calcd. for: C_32_H_34_N_4_O_4_S: (570.23): C, 67.35; H, 6.00; N, 9.82%. 200.42 Found: C, 67.51; H, 6.09; N, 9.74%.

#### 7-Acetyl-1-amino-*N*-(4-chlorophenyl)−5,8-dimethyl-8-hydroxy-6-(3-nitrophenyl)−6,7,8,9-tetrahydrothieno[2,3-c]isoquinoline-2-carboxamide (7c)

It was obtained by reaction of **2a** with *N*-(4-chlorophenyl)−2-chloroacetamide in the presence of sodium carbonate. Yield: 94%; m.p.: 293–294 °C. Anal. The FT-IR spectrum of compound **7c**: 3417, 3383, 3314 cm^−1^ for (O–H, NH_2_, N–H); 3095 cm^−1^ for (C–H, sp^2^); 2967, 2916 cm^−1^ for (C–H, sp^3^); 1706 cm^−1^ for (C=O, acetyl); 1622 cm^−1^ for (C=O, amide) .^1^H NMR spectrum of compound **7c** in (400 MHz, DMSO-d_6_) show signals at δ: 9.56 (s, 1H, NH), 8.06–8.08 (m, 1H, 1Ar-H), 7.74–7.84 (m, 3H, 3Ar-H), 7.57 – 7.58 (d, 2H, 2Ar-H), 7.51–7.55 (t, 2H, NH_2_), 7.13–7.39 (m, 2H, Ar–H), 4.85–4.88(t, J = 12 Hz, 2H, OH, C^9^H), 3.64–3.67 (s, 1H, C^7^H), 3.40–3.44 (d, J = 12 Hz, 1H, C^9^H), 2.93–2.95 (d, J = 8 Hz, 1H, C^6^H), 2.21 (s, 3H,CO CH_3_), 2.04 (s, 3H, CH_3_), 1.33 (s, 3H, CH_3_). ^13^C NMR spectrum of compound **7c** (DMSO-d6) show 25 environments of carbon as expected at δ: 209.42, 164.35, 158.33, 156.65, 149.62, 147.92, 147.04, 142.94, 135.07, 130.10, 128.27, 128.23, 126.96, 122.95, 122.65, 122.41, 121.51, 96.41, 67.14, 65.88, 42.8, 41.99, 31.17, 27.94, 24.74. Anal. Calcd. for C_28_H_25_ClN_4_O_5_S (564.12): C, 59.52; H, 4.46; N, 9.92%. Found: C, 59.3; H, 5.01; N, 9.90%.

#### 2-[(7-Acetyl-4-cyano-1,6-dimethyl-6-hydroxy-8-(3-nitrophenyl)−5,6,7,8-tetrahydro-isoquinolin-3-yl)thio]-*N*-(naphthalen-1-yl)acetamide (8a)

It was obtained by reaction of compound **2a** with *N*-(1-naphthyl)−2-chloroacetamide (IV) Yield: 86%; m.p.: 237–238 °C. The FT-IR spectrum of compound **8a**: 3527 cm^−1^ for (O–H); 3401 cm^−1^ for (N–H); 3085 cm^−1^ for (C–H, sp^2^); 2970, 2928 cm^−1^ for (C–H, sp^3^); 2214 cm^−1^ for (C≡N); 1702 cm^−1^ for (C=O, acetyl); 1665 cm^−1^ for (C=O, amide)0.1597 cm^−1^ for (C=N). ^1^H NMR spectrum of compound **8a** in (400 MHz, DMSO-d_6_) show signals at δ: 10.19 (s, 1H, NH); 7.37–8.08 (m, 11H, ArH); 5.00–5.01 (s, 1H, OH); 4.75–4.76 (d, J = 8 Hz, 1H, C^8^H); 4.27 (d, 2H, SCH_2_); 3.25–3.26 (d, J = 4 Hz, 1H, C^5^H), 2.87–2.95 (m, 2H: C^7^H and C^5^H), 2.14–2.15 (d, J = 12 Hz, 3H, COCH_3_); 1.96–1.97 (m, J = 12 Hz, 3H, CH_3_); 125 (s, 3H, CH_3_). Anal. Calcd. for C_33_H_30_N_4_O_4_S (578.69): C, 68.49; H, 5.23; N, 9.68%. Found: C, 65.88; H, 4.75; N, 9.41%.

#### 2-[(7-Acetyl-4-cyano-1,6-dimethyl-6-hydroxy-8-(4-nitrophenyl)−5,6,7,8-tetrahydro-isoquinolin-3-yl)thio]-*N*-(naphthalen-1-yl)acetamide (8b)

It was synthesized by reaction of **2b** with *N*-(1-naphthyl)−2-chloroacetamide (IV). Yield: 93%; m.p.: 230–232 °C. The FT-IR spectrum of compound **8b**: 3604–3489 cm^−1^ for (O–H); 3356 cm^−1^ for (N–H); 3252(C–H, sp^2^); 2971, 2927 cm^−1^ for (C–H, sp^3^); 2218 cm^−1^ for (C≡N); 1705–1686 cm^−1^ for (C=Oamide, C=O acetyl). ^1^H NMR spectrum of compound **8b** in (500 MHz, DMSO-d_6_) show signals at δ: (500 MHz,) δ 10.23 (s, 1H, NH), 7.74–7.90 (m, 5H, Ar–H), 7.54–7.60 (m, 8H, Ar–H), 4.78–4.80 (d, J = 10.4 Hz, 1H, OH), 4.33–4.47 (m, 2H, SCH_2_), 3.46–3.47 (dd, J = 14.1, 7.0 Hz, 1H, C^8^H), 3.32–3.35 (d, J = 17.1 Hz, 1H, C^5^H), 2.95–2.98 (m, 2H, C^7^H and C^5^H), 2.17 (S, 3H, CH_3_), 1.99–2.00 (d, J = 4 Hz, 3H, CH_3_), 1.30–1.31 (d, J = 4 Hz, 3H, CH_3_).^13^C NMR spectrum of compound **8b** (DMSO-d6) show environments of carbon as expected at δ: 208.5, 166.87, 165.62, 160.63, 157.87, 151.84, 150. 08, 146.09, 133.69, 131.8, 128.16, 126.12, 126.00, 122.53, 122.52, 121.88, 121.88, 115.1, 104.19, 67.40, 65.7, 61.96, 56.06, 43.35, 31.17, 27.51, 24.63, 18.53. Anal. Calcd. for C_33_H_30_N_4_O_4_S (578.69): C, 68.49; H, 5.23; N, 9.68%. Found: C, 66.33; H, 4.92; N, 9.68%.

#### 7-Acetyl-1-amino-*N*-(naphthalen-1-yl)−5,8-dimethyl-8-hydroxy-6-(3-Nitro phenyl)−6,7,8,9-tetrahydrothieno[2,3-c]isoquinoline-2-carboxamide (9a).

It was obtained by cyclization of compound **8a** Yield: 96%; m.p.: 290–293 °C. Color: yellow light powder. Recrystallized from ethanol. The FT-IR spectrum of compound **9a**: 3404 cm^−1^ for (O–H, NH_2_, NH); 2922 cm^−1^ for (C–H, sp^2^); 2852 cm^−1^ for (C–H, sp^3^); 1707 cm^−1^ for (C=O, acetyl). ^1^H NMR spectrum of compound **9a** in (90 MHzCDCl3): δ 9.75 (1H, NH), 7.20–8.30 (11H, Ar–H), 6.70 (2H, NH_2_), 3.6 (1H, OH), 2.70 (1H, C^6^H), 2.40 (1H, C^9^H) 2.10 (1H, C^9^H), 1.90 (1H, C^7^H), 1.35 (6H, CH_3_,COCH_3_), 0.95 (3H, CH_3_(. Anal. Calcd for C_33_H_30_N_4_O_4_S (578.69): C, 68.49; H, 5.23; N, 9.68%. Found: C, 69.1; H, 5.18; N, 9.52%.

#### 7-Acetyl-1-amino-*N*-(naphthalen-1-yl)−5,8-dimethyl-8-hydroxy-6-(4-Nitro phenyl)−6,7,8,9-tetrahydrothieno[2,3-c]isoquinoline-2-carboxamide (9b).

It was obtained by cyclization of compound **8b** Yield: 89%; m.p.: 286–289 °C. Color: yellow light powder. Recrystallized from ethanol. The FT-IR spectrum of compound **9b**: 3481 cm^−1^ for (O–H, NH_2_, NH); 2956, 2924 cm^−1^ for (C–H, sp^2^); 2853 cm^−1^ for (C–H, sp^3^); 1706 cm^−1^ for (C=O, acetyl). ^1^H NMR spectrum of compound **9b** in (90 MHzCDCl3): δ 3.3 (s, 1H, NH), 7.00–8.40 (11H, Ar–H), 6.70 (br s, 2H, NH_2_), 3.8 (s, 1H, OH), 2.20–22.30 (2H, C^9^H, C^6^H), 2.00 (1H, C^9^H), 1.7 (1H, C^7^H), 1.40 (s, 3H, CH_3_, at C-5), 1.20 (s, 3H, COCH_3_), 1.00 (s, 3H, CH_3_). Anal. Calcd for C_33_H_30_N_4_O_4_S (578.69): C, 68.49; H, 5.23; N, 9.68%. Found: C, 69.33; H, 5.25; N, 9.59%.

### Synthesis of 7-Acetyl-1-amino-*N*-(benzthiazol-2-yl)−5,8-dimethyl-8-hydroxy-6-(3-nitrophenyl)−6,7,8,9-tetrahydrothieno[2,3-c]isoquinoline-2-carboxamide (10)

It was obtained by reaction of **2a** (10 mmol) with *N*-(benzthiazol-2-yl)−2-chloroacetamide (**V**) (10 mmol), and sodium acetate trihydrate (1.50 g, 11 mmol) in ethanol (100 mL) was refluxed for one hour. The solid that formed after cooling was collected and then recrystallized from ethanol to give white crystals of compound **10** directly.

Yield: 97%; m.p.:300–305 °C. FT-IR: 3431, 3319 cm^−1^ for (O–H, NH_2_, NH); 2973 cm^−1^ for (C–H, sp^2^); 1707 cm^−1^ for (C=O, acetyl); ^1^H NMR (400 MHz, DMSO-d_6_) showed signals at δ: 7.22–8.08 (m, 11H, NH_2_, 11Ar-H), 4.86–4.88 (s, 2H, OH, C^9^H), 3.65–3.68 (d, J = 12 Hz, 1H, C^6^H), 3.41–3.44 (d, J = 4 Hz, 1H, C^7^H), 2.90 (s, 1H, C^9^H), 2.17 (s, 3H, COCH_3_), 1.93 (s, 3H, CH_3_), 1.34 (s, 3H, CH_3_).^13^C NMR of compound **10** (dmso) δ 209.47, 158.18, 157.84, 147.91, 147.12, 142.94, 135.08, 130.10, 127.91.67, 123.27, 123.1, 122.4, 121.50, 67.16, 65.93, 42.90, 41.97, 31.17, 27.98, 24.74, 18.53. Anal. Calcd. For C_29_H_25_N_5_O_5_S_2_ (587.67)%: C, 59.27; H, 4.29; N, 11.92; O, 13.61; S, 10.91., Found C, 58.07; H, 4.35; N, 12.00.

### Cytotoxicity against human cancer cell lines

Some of the synthesized compounds were tested as anticancer activity for the IC_50_ against two cell lines **HEPG2** and **MCF7** cells according to method [52, 81]. All cell lines were obtained from national cancer institute, Cairo—Egypt.

### Cell cycle analysis

The cell cycle arrests of compound **3** against **HEPG2** was carried out according to Abcam method (code ab139418), (www.abcam.co.jp) [52, 82, 83].

### Annexin-V FITC apoptosis assay

The Annexin-V FITC apoptosis assay of compounds **3** against **HEPG2** was performed according to (BioVision Research Products (code k101-25). (www.biovision.com) [52, 84, 85].

### Molecular docking Materials and Methods for RET Enzyme

Molecular docking studies were performed in **(I Mole Lab for Bioinformatics-Cairo**).

#### Ligand preparation

The retrieved Ligands structures were subjected to energy minimization using the Avogadro 1.2.0 software with the MMFF94 force field [86].

#### Protein target selection and preparation for RET enzyme

The selected target was the RET tyrosine kinase receptor was retrieved form data base (UniProt ID: Q9UMQ4). The protein structures were prepared using AutoDock Tools 1.5.7 [87].

#### Binding site identification for RET enzyme

The potential binding pockets on the selected protein targets were identified using the CB-Dock 2 webserver [88].

#### Molecular Docking for RET enzyme

Molecular docking studies were performed according to method [89].

#### Data analysis and visualization for RET enzyme

The results from molecular docking prediction were analyzed using appropriate computational tools and software. The visualization of protein–ligand interactions and the generation of figures were performed using Biovia 2020.

#### Ligand retrieval for HSP90 enzyme

Ligands Were retrieved from Pubchem using CID (Onalespib: 11955716) using SDF format, then all ligands were energy minimized using Avogadro 1.2.0 [90] software using MMFF94 force field due to Organic nature of compounds and saved using suitable Format.

#### Protein preparation for HSP90 enzyme

Proteins structure for, HSP90 enzyme was retrieved form data base uniprot ID (P08238), then protein prepared according to [87].

#### Molecular docking and visualization for HSP90 enzyme

Molecular docking simulation were performed using Autodock vina [91].

## Conclusion

In this article, we synthesized and characterized a new tetrahydroisoquniolines compounds. Some of the synthesized compounds were examined for their anticancer activity towards **HEPG2** and **MCF7** cell lines. They showed high anticancer activities. Moreover, the cell cycle arrest and apoptosis induction of the one compounds was studied. Compound **3** caused cell cycle arrest of **HEPG2** cell line at G2/M phase and caused high increase in the early and late apoptosis and necrosis. Furthermore, we applied the molecular docking study for compound **8a** and it showed significant inhibition for **RET** enzyme while compound **3** exhibited high binding ability for **HSP90** enzyme. This ensure the pervious article studies reported that tetrahydroisoquinoline compounds can be used as enzyme inhibitors.

## Supplementary Information

Supplementary Material 1

## Data Availability

All data generated or analyzed during this study are in this published article and supplementary information and you can ask the corresponding author for any additional information’s.
