# Supplementary material for: New tetrahydroisoquinolines bearing nitrophenyl group targeting HSP90 and RET enzymes: synthesis, characterization and biological evaluation
Source: BMC Chem. 2025 Feb 21;19(1):46. doi: 10.1186/s13065-025-01399-0 (PMC11846289; doi:10.1186/s13065-025-01399-0)
Supplement: Supplementary file 1 — Supplementary Material 1 [file 13065_2025_1399_MOESM1_ESM.docx]

**New Tetrahydroisoquinolines Bearing Nitrophenyl Group Targeting HSP90 and RET Enzymes: Synthesis, Characterization and Biological Evaluation.**

**Etify A. Bakhite^*1^, Reda Hassanien^2^, Nasser Farhan^2^, Eman M. Sayed^*2^, Marwa Sharaky ^3^**

^1^Chemistry Department, Faculty of Science, Assuit University, 71516 Assiut, Egypt.

^2^Chemistry Department, Faculty of Science, New Valley University, 72511 El-Kharja, Egypt

^3^ Pharmacology Unit - Cancer Biology Department - National Cancer Institute -  Cairo University-12613 EL- Gize, EGYPT**.**

**Figure S1**: FT**-**IR spectrum of the start 7-acetyl-6-hydroxy-1,6-dimethyl-8-(3-nitrophenyl)-3-thioxo-2,3,5,6,7,8-hexahydroisoquinoline-4-carbonitrile **(2a)**


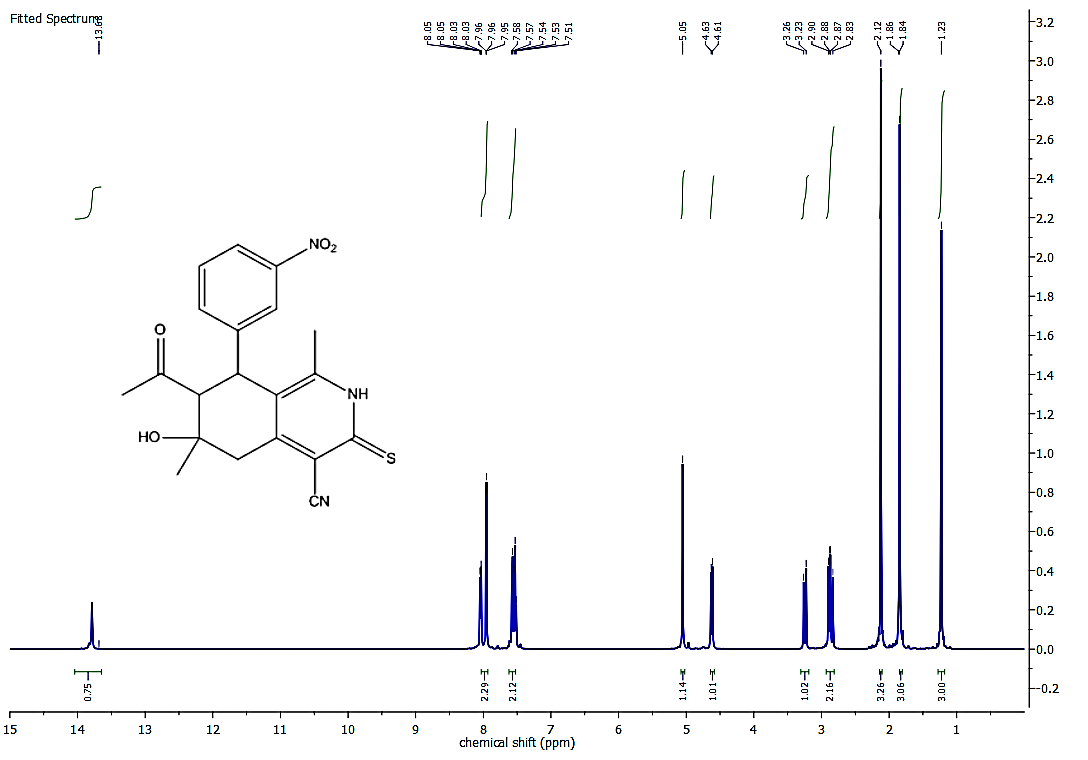


**Figure S 2:** ^1^H NMR spectrum of the start 7-acetyl-6-hydroxy-1,6-dimethyl-8-(3-nitrophenyl)-3-thioxo-2,3,5,6,7,8-hexahydroisoquinoline-4-carbonitrile **(2a)**

**Figure S3**: FT**-**IR spectrum of the start 7-acetyl-6-hydroxy-1,6-dimethyl-8-(4-nitrophenyl)-3-thioxo-2,3,5,6,7,8-hexahydroisoquinoline-4-carbonitrile **(2b)**


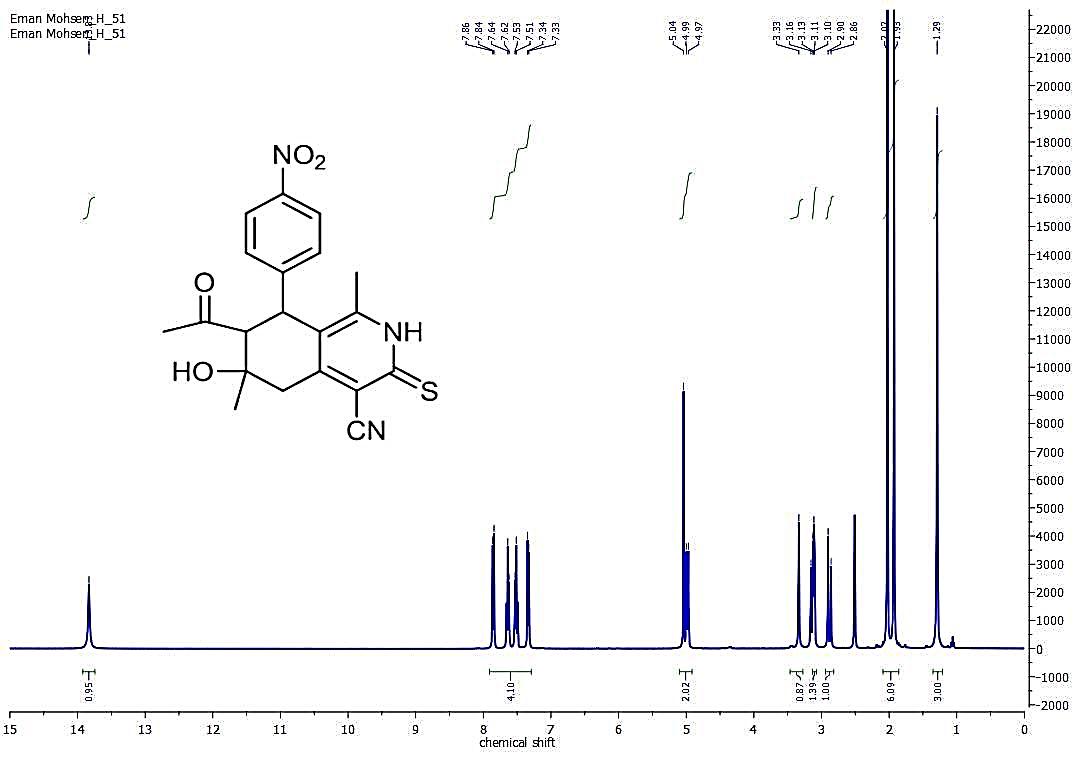


**Figure S4**: ^1^HNMR spectrum of the start 7-acetyl-6-hydroxy-1,6-dimethyl-8-(4-nitrophenyl)-3-thioxo-2,3,5,6,7,8-hexahydroisoquinoline-4-carbonitrile **(2b)**

**Figure S5**: FT**-**IR spectrum of the start 7-acetyl-3-(methylthio)-6-hydroxy-1,6-dimethyl-8-(3-nitrophenyl)-5,6,7,8-tetrahydroisoquinoline-4-carbonitrile **(3).**


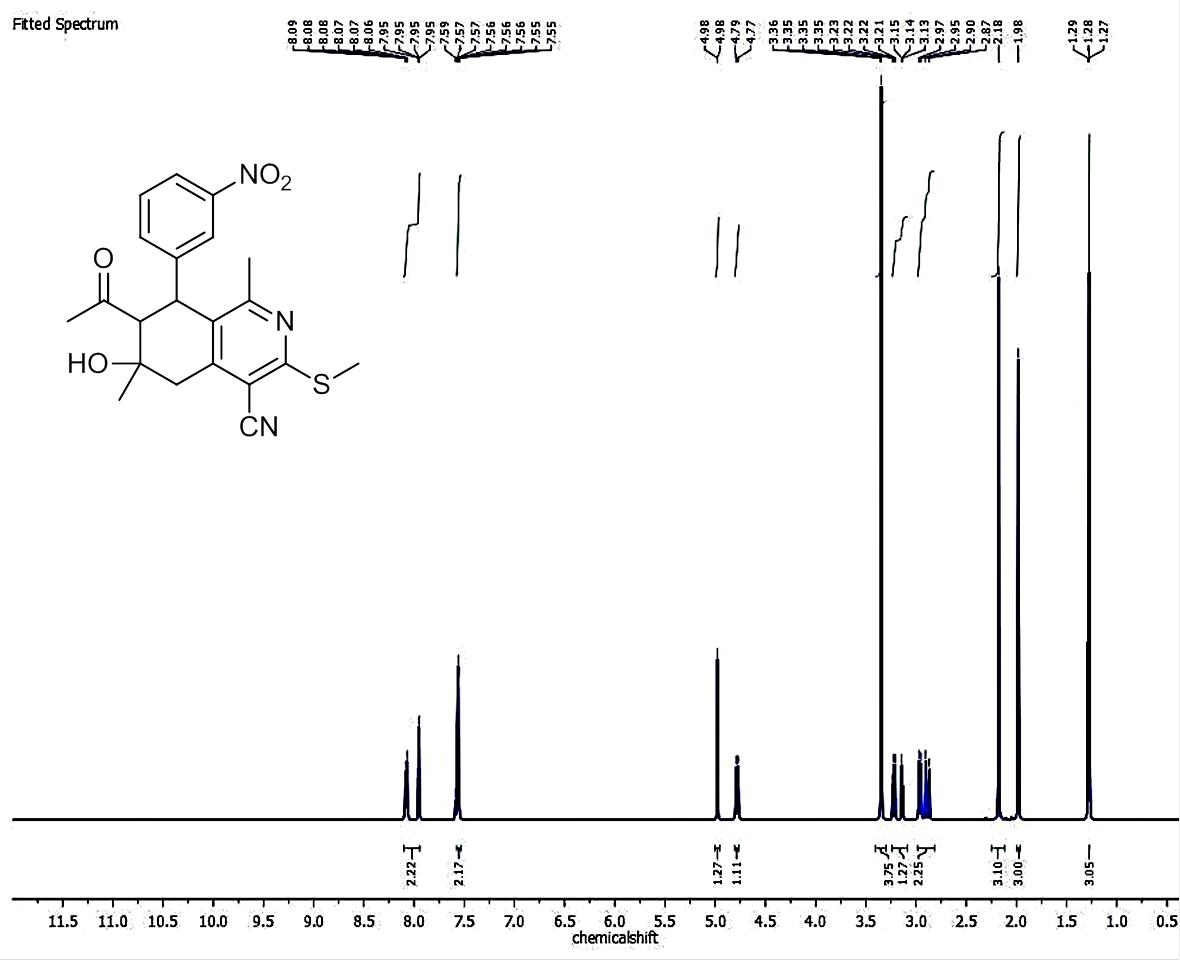


**Figure S 6**: ^1^H NMR spectrum of the start 7-acetyl-3-(methylthio)-6-hydroxy-1,6-dimethyl-8-(3-nitrophenyl)-5,6,7,8-tetrahydroisoquinoline-4-carbonitrile **(3).**


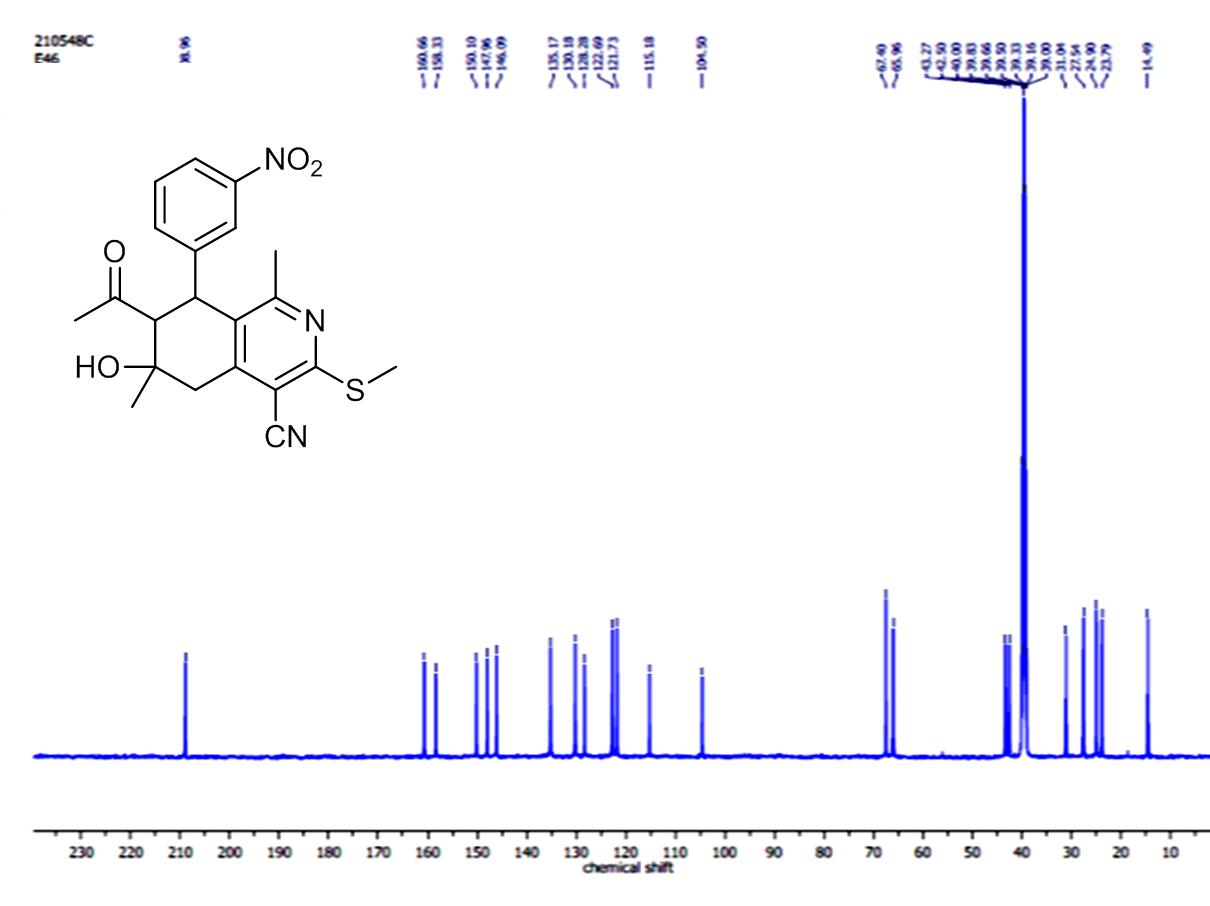


**Figure S7**: C13 spectrum of the start 7-acetyl-3-(methylthio)-6-hydroxy-1,6-dimethyl-8-(3-nitrophenyl)-5,6,7,8-tetrahydroisoquinoline-4-carbonitrile **(3).**

**Figure S8**: FT**-**IR spectrum of the ethyl 2-((7-acetyl-4-cyano-6-hydroxy-1,6-dimethyl-8-(3-nitrophenyl)-5,6,7,8-tetrahydroisoquinolin-3-yl)thio)acetate **(4)**.


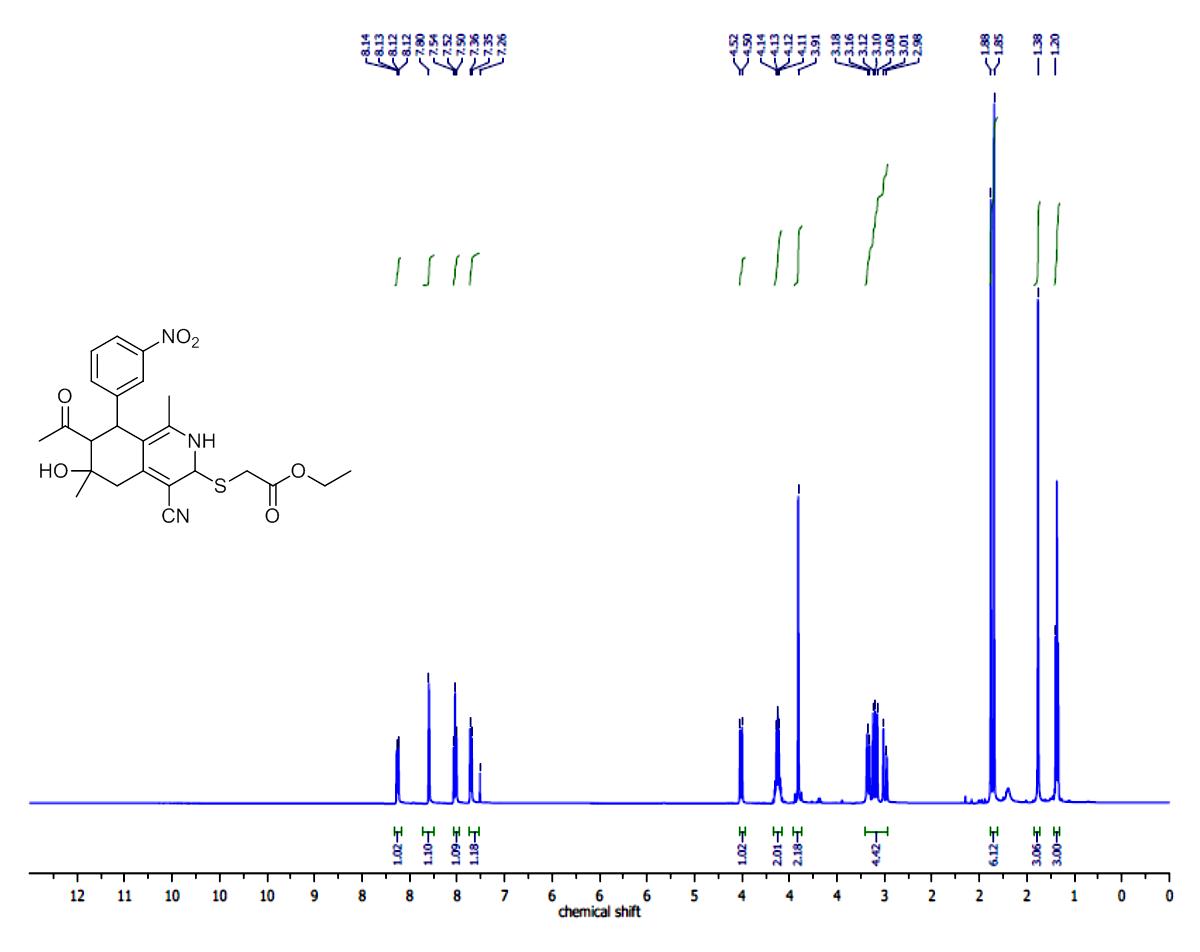


**Figure S9**: ^1^H NMR spectrum of the ethyl 2-((7-acetyl-4-cyano-6-hydroxy-1,

6-dimethyl-8-(3-nitrophenyl)-5,6,7,8-tetrahydroisoquinolin-3-yl)thio)acetate **(4)**.


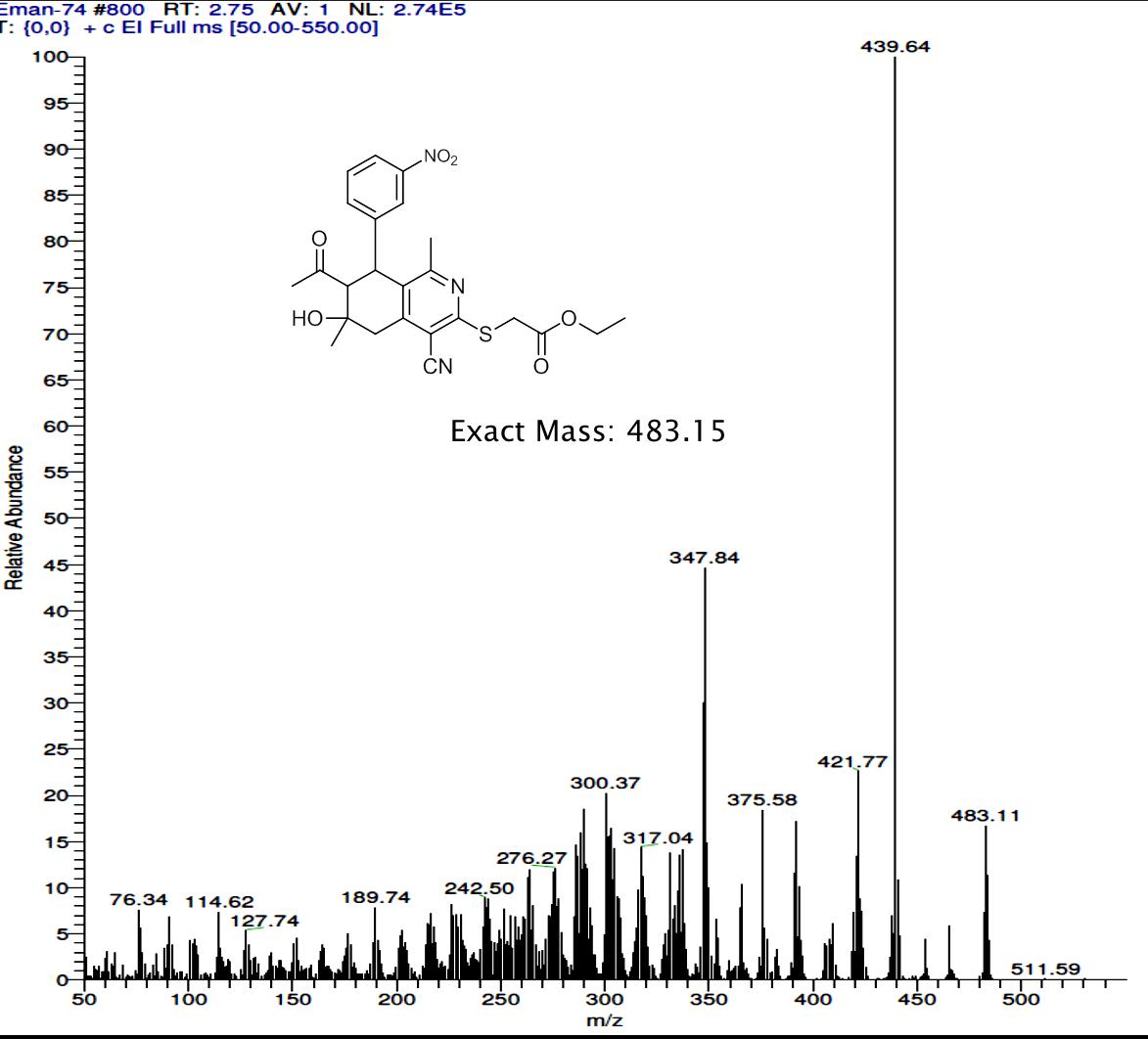


**Figure S10**: Mass spectrum of the ethyl 2-((7-acetyl-4-cyano-6-hydroxy-1,6-

dimethyl-8-(3-nitrophenyl)-5,6,7,8-tetrahydroisoquinolin-3-yl)thio)acetate **(4)**.

**Figure S**11: FT-IR Spectrum of the 7-acetyl-3-((cyanomethyl)thio)-6-hydroxy-6-methyl-8-(4-nitrophenyl)-5,6,7,8-tetrahydroisoquinoline-4-carbonitrile **(5).**

**
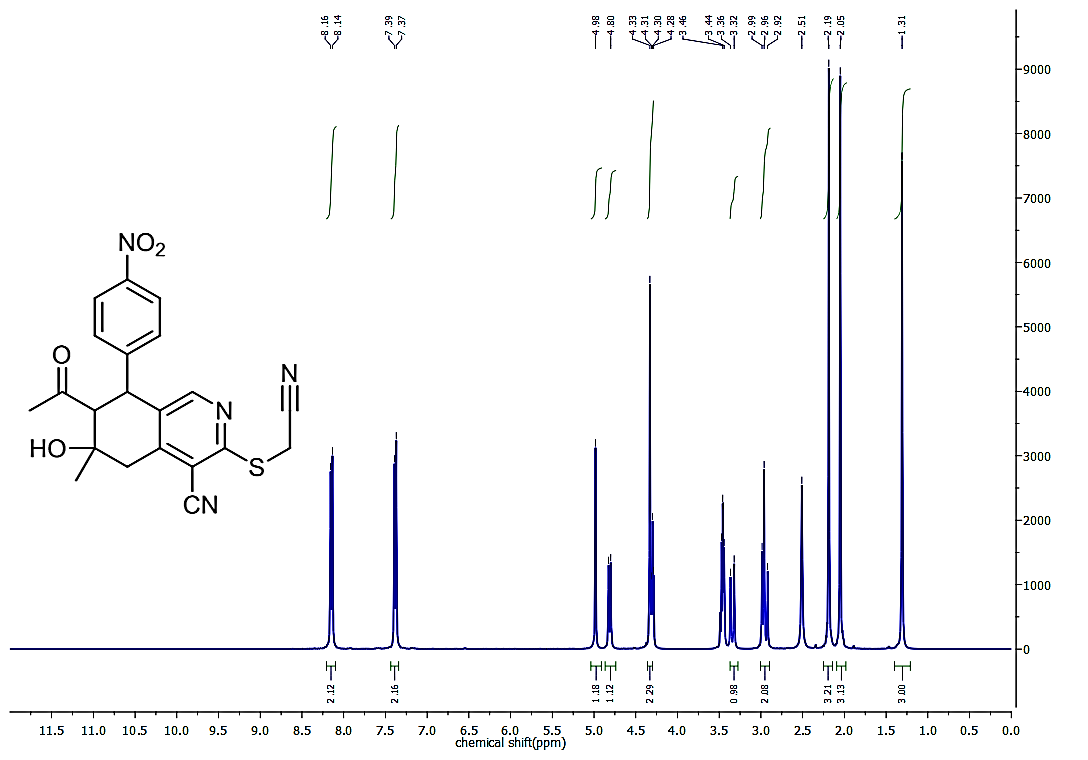
**

**Figure S12**: ^1^H NMR Spectrum of the 7-acetyl-3-((cyanomethyl)thio)-6-hydroxy-6-methyl-8-(4-nitrophenyl)-5,6,7,8-tetrahydroisoquinoline-4-carbonitrile **(5).**


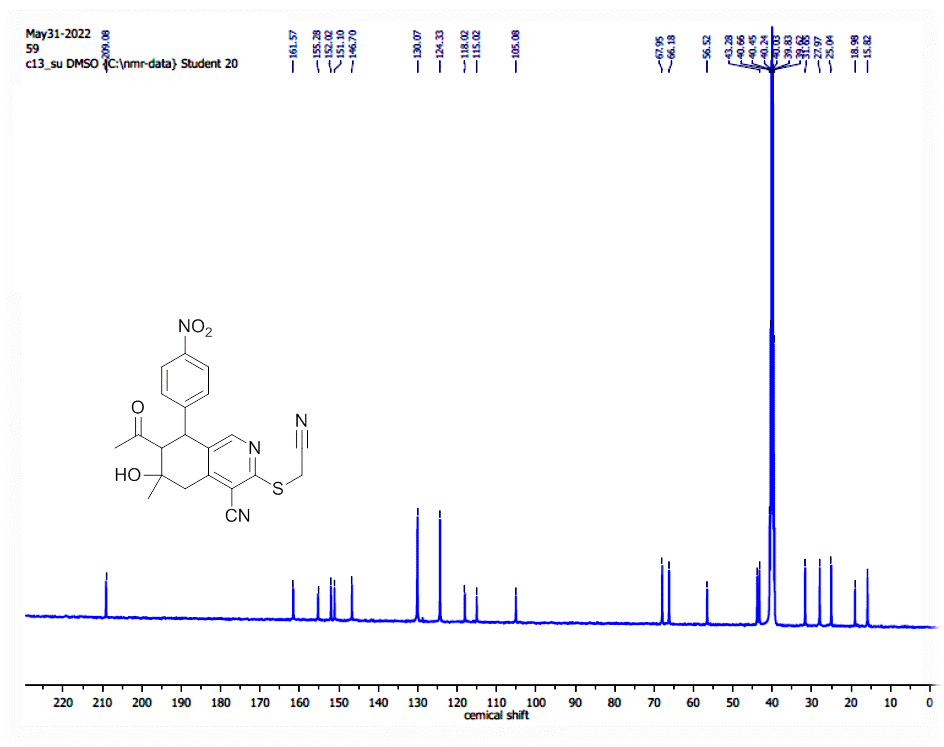


**Figure S**13: ^13^ CNMR Spectrum of the 7-acetyl-3-((cyanomethyl)thio)-6-hydroxy-6-methyl-8-(4-nitrophenyl)-5,6,7,8-tetrahydroisoquinoline-4-carbonitrile **(5)**

**Figure S14**: FT**-**IR spectrum of the 2-((7-acetyl-4-cyano-6-hydroxy-1,6-dimethyl-8-(3-nitrophenyl)-5,6,7,8-tetrahydroisoquinolin-3-yl)thio)-N-(4-acetylphenyl)acetamide **(6a)**.

**
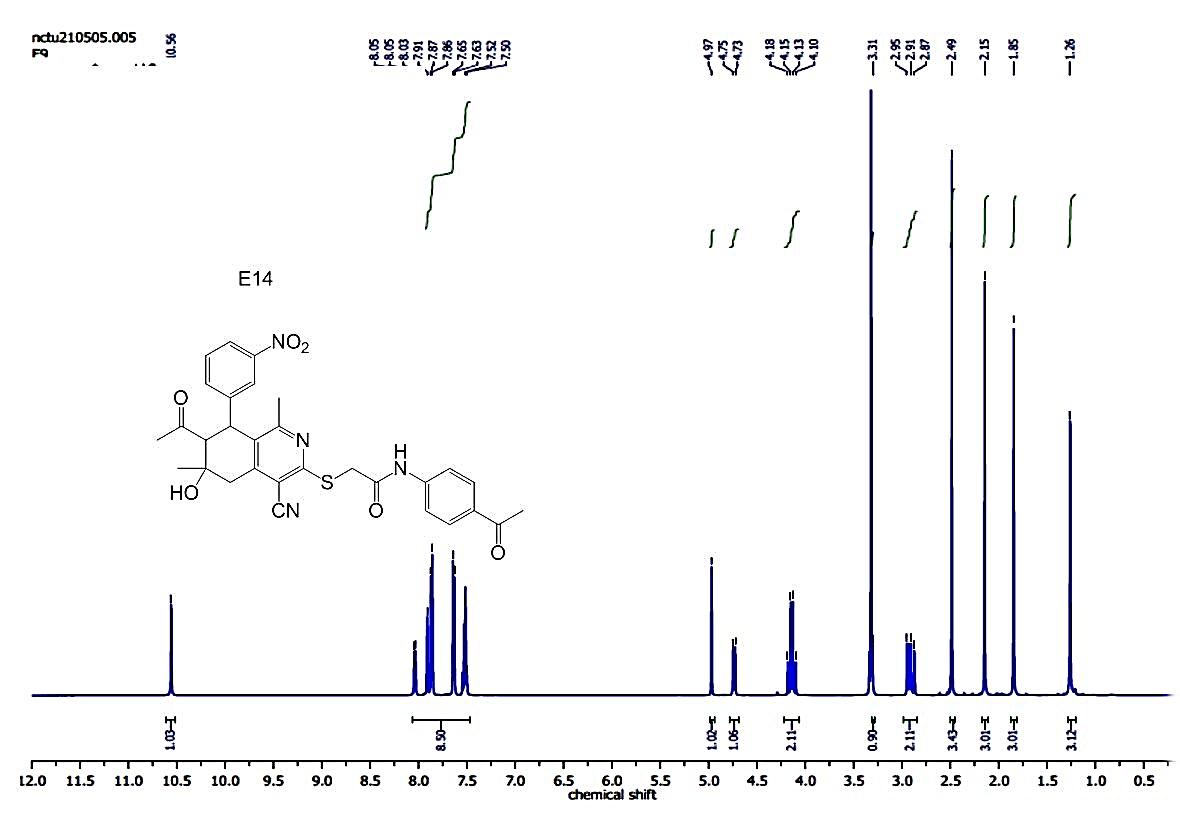
 Figure S15**: ^1^H NMR spectrum of the 2-((7-acetyl-4-cyano-6-hydroxy-1,6-dimethyl-8-(2-nitrophenyl)-5,6,7,8-tetrahydroisoquinolin-3-yl)thio)-N-(4-acetylphenyl)acetamide**(6a)**.


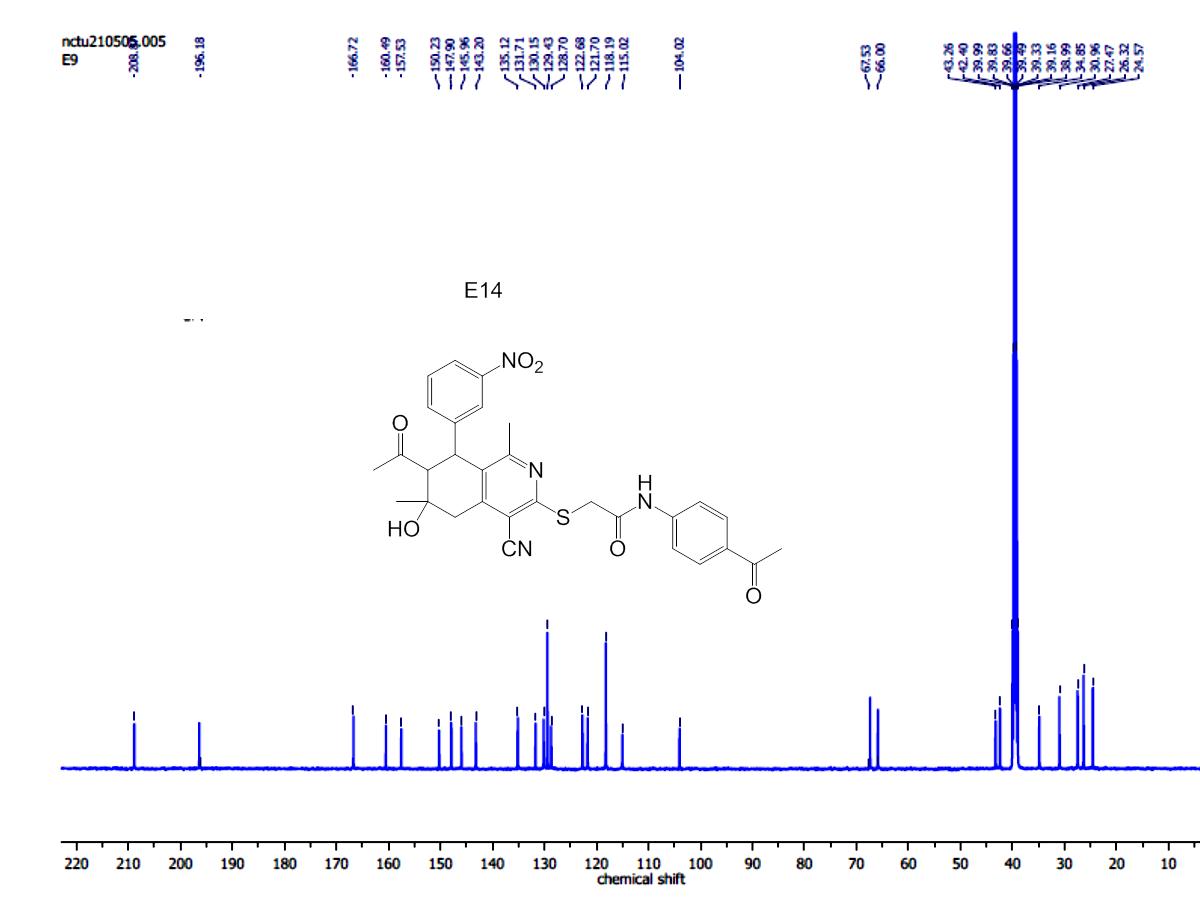


**Figure S16**: C^13^ spectrum of the 2-((7-acetyl-4-cyano-6-hydroxy-1,6-dimethyl-8-(3-nitrophenyl)-5,6,7,8-tetrahydroisoquinolin-3-yl)thio)-N-(4-acetylphenyl)acetamide **(6a)**.

**Figure S17**: FT**-**IR spectrum of the 2-((7-acetyl-4-cyano-6-hydroxy-1,6-dimethyl-8-(3-nitrophenyl)-5,6,7,8-tetrahydroisoquinolin-3-yl)thio)-N-(4-acetylphenyl)acetamide **(6b)**.


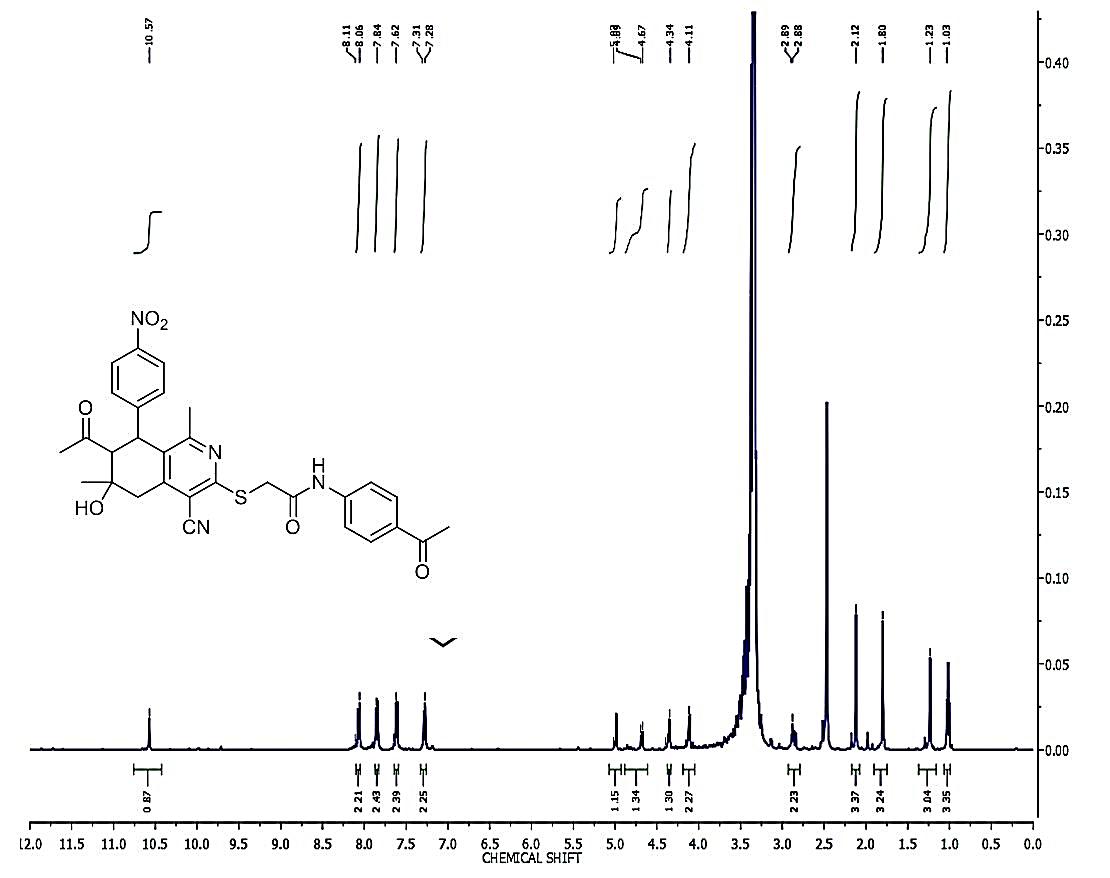
 **Figure S18**: ^1^H NMR spectrum of the 2-((7-acetyl-4-cyano-6-hydroxy-1,6-dimethyl-8-(3-nitrophenyl)-5,6,7,8-tetrahydroisoquinolin-3-yl)thio)-N-(4-acetylphenyl)acetamide **(6b**).

**Figure S19:**  FT**-**IR spectrum of 2-((7-acetyl-4-cyano-6-hydroxy-1,6-dimethyl-8-(4-nitrophenyl)-5,6,7,8-tetrahydroisoquinolin-3-yl)thio)-N-(4-chlorophenyl)acetamide  **(6c)**.

**
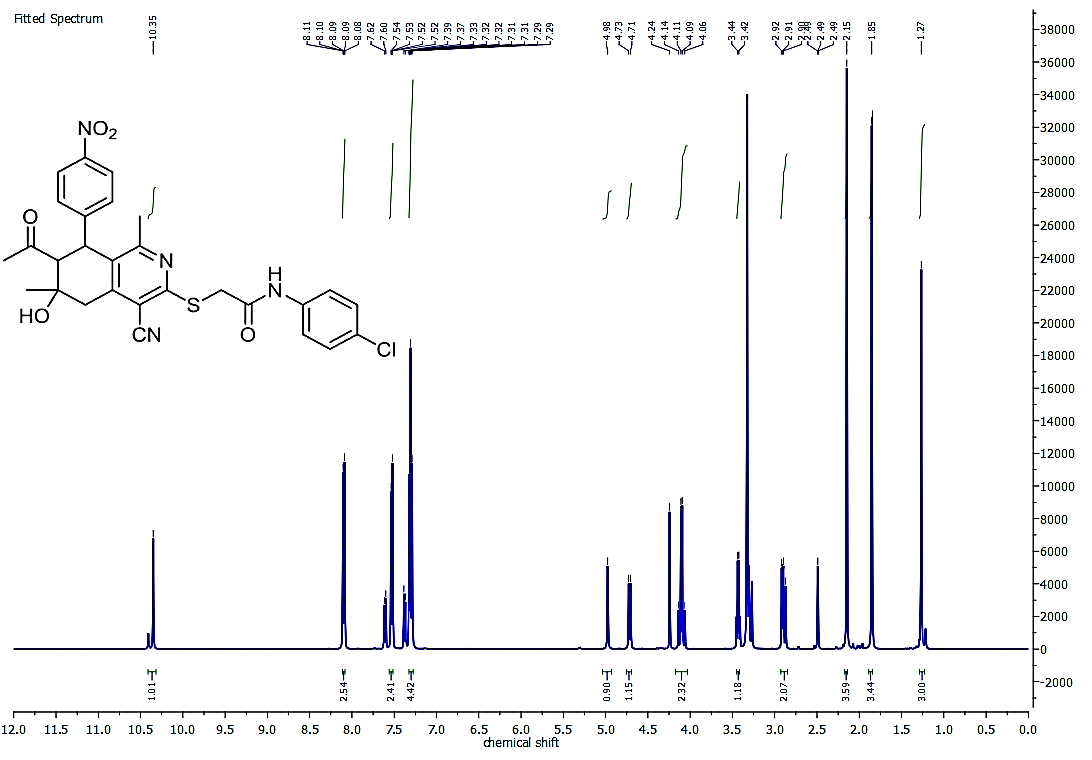
**

**Figure S20:**  ^1^H NMR spectrum of 2-((7-acetyl-4-cyano-6-hydroxy-1,6-dimethyl-8-(4-nitrophenyl)-5,6,7,8-tetrahydroisoquinolin-3-yl)thio)-N-(4-chlorophenyl)acetamide  **(6c)**.


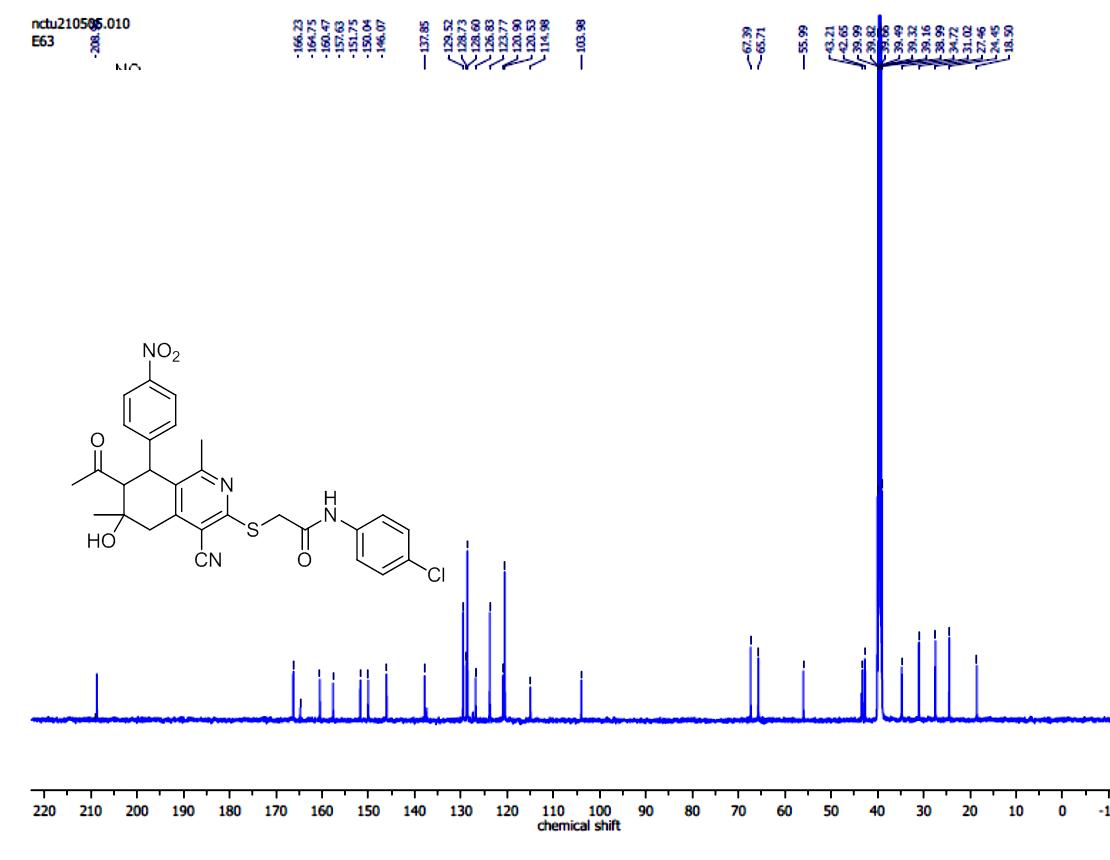


**Figure S21: ^13^C** spectrum of 2-((7-acetyl-4-cyano-6-hydroxy-1,6-dimethyl-8-(4-nitrophenyl)-5,6,7,8-tetrahydroisoquinolin-3-yl)thio)-N-(4-chlorophenyl)acetamide  **(6c)**.

**Figure S22**: FT**-**IR spectrum of the 7-acetyl-N-(4-acetylphenyl)-1-amino-8-hydroxy-5,8-dimethyl-6-(3-nitrophenyl)-6,7,8,9-tetrahydrothieno[2,3-c]isoquinoline-2-carboxamide **(7a)**.

#
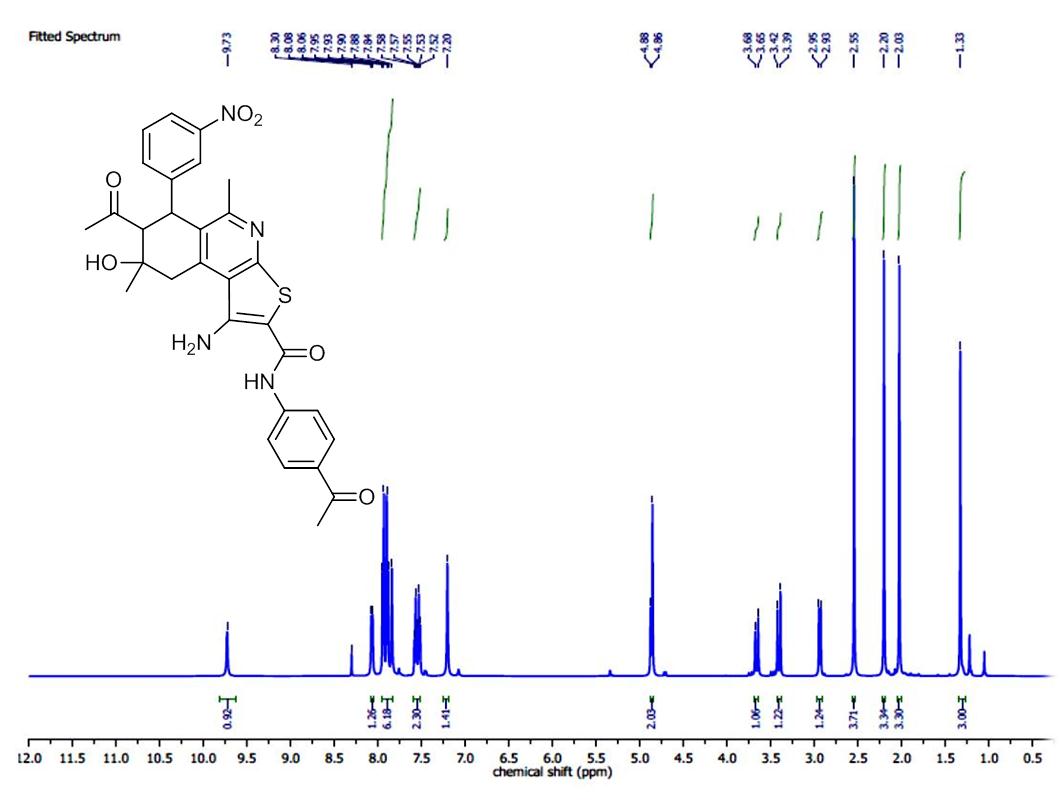


**Figure S23:** ^1^H NMR spectrum of the 7-acetyl-N-(4-acetylphenyl)-1-amino-8-hydroxy-5,8-dimethyl-6-(3-nitrophenyl)-6,7,8,9-tetrahydrothieno[2,3-c]isoquinoline-2-carboxamide **(7a)**

**
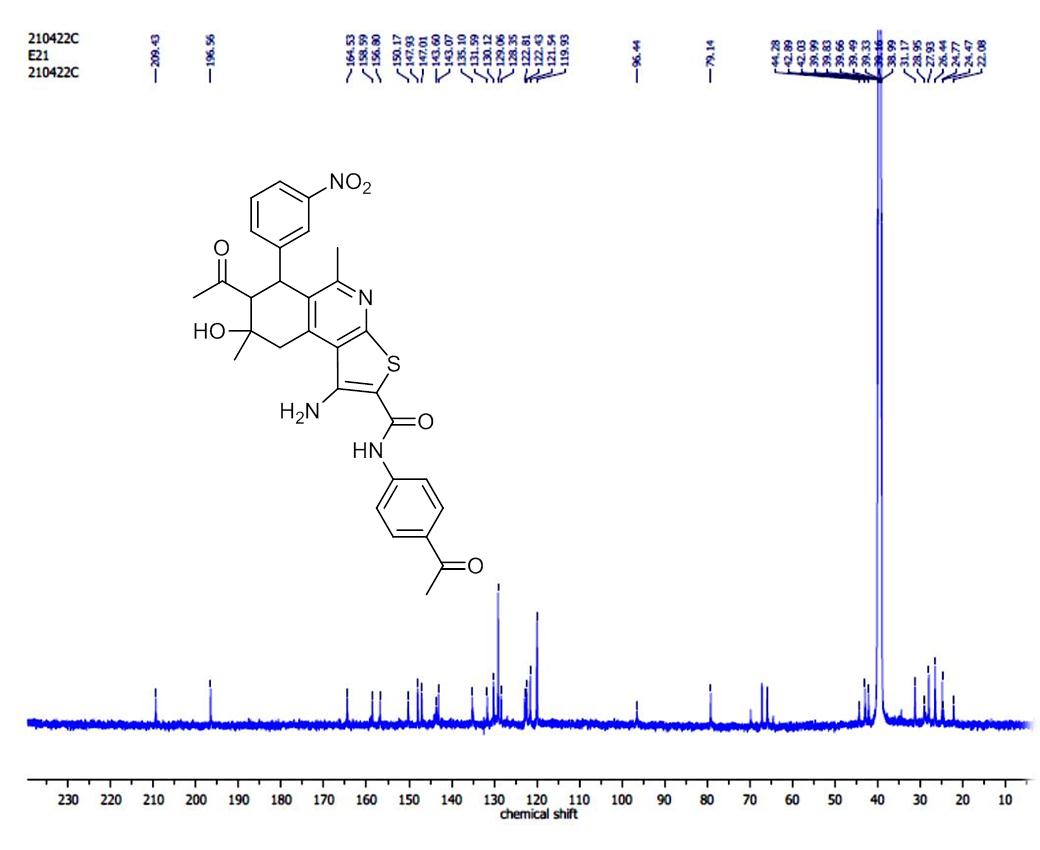
**

**Figure 24**: ^13^C NMR Spectrum of the 7-acetyl-N-(4-acetylphenyl)-1-amino-8-hydroxy-5,8-dimethyl-6-(3-nitrophenyl)-6,7,8,9-tetrahydrothieno[2,3-c]isoquinoline-2-carboxamide **(7a).**

**Figure S25**: FT**-**IR spectrum of the 7-acetyl-N-(4-acetylphenyl)-1-amino-8-hydroxy-5,8-dimethyl-6-(4-nitrophenyl)-6,7,8,9-tetrahydrothieno[2,3-c]isoquinoline-2-carboxamide **(7b)**.

**Figure S26**: ^1^HNMR spectrum of the 7-acetyl-N-(4-acetylphenyl)-1-amino-8-hydroxy-5,8-dimethyl-6-(4-nitrophenyl)-6,7,8,9-tetrahydrothieno[2,3-c]isoquinoline-2-carboxamide **(7b)**

**Figure S27**: **^13^C**NMR spectrum of the 7-acetyl-N-(4-acetylphenyl)-1-amino-8-hydroxy-5,8-dimethyl-6-(4-nitrophenyl)-6,7,8,9-tetrahydrothieno[2,3-c]isoquinoline-2-carboxamide **(7b)**.

**Figure S**28: FT-IR Spectrum of 7-Acetyl-1-amino-*N*-(4-chlorophenyl)-5,8-dimethyl-8-hydroxy-6-(3-nitrophenyl)-6,7,8,9-tetrahydrothieno[2,3-c]isoquinoline-2-carboxamide **(7C).**


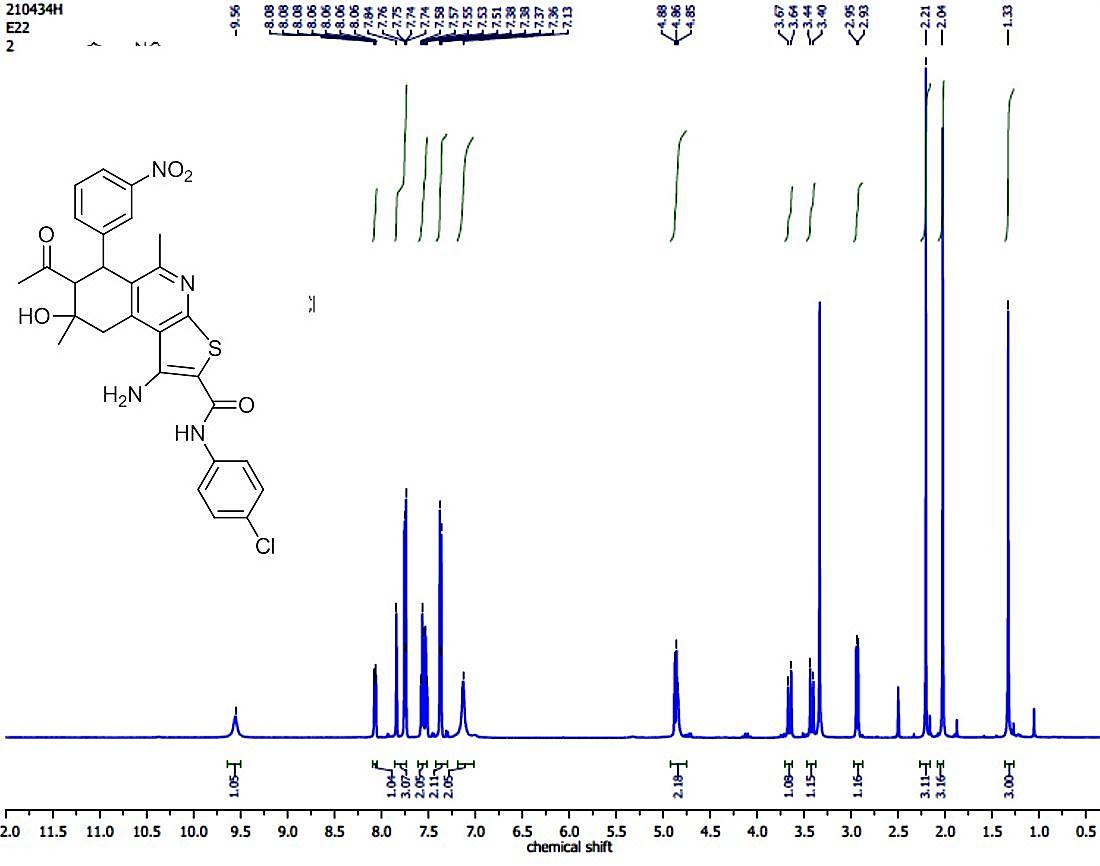


**Figure S**29:^1^H NMR Spectrum of 7-Acetyl-1-amino-*N*-(4-chlorophenyl)-5,8-dimethyl-8-hydroxy-6-(3-nitrophenyl)-6,7,8,9-tetrahydrothieno[2,3-c]isoquinoline-2-carboxamide **(7C**) in DMSO-*d_6_*.

**
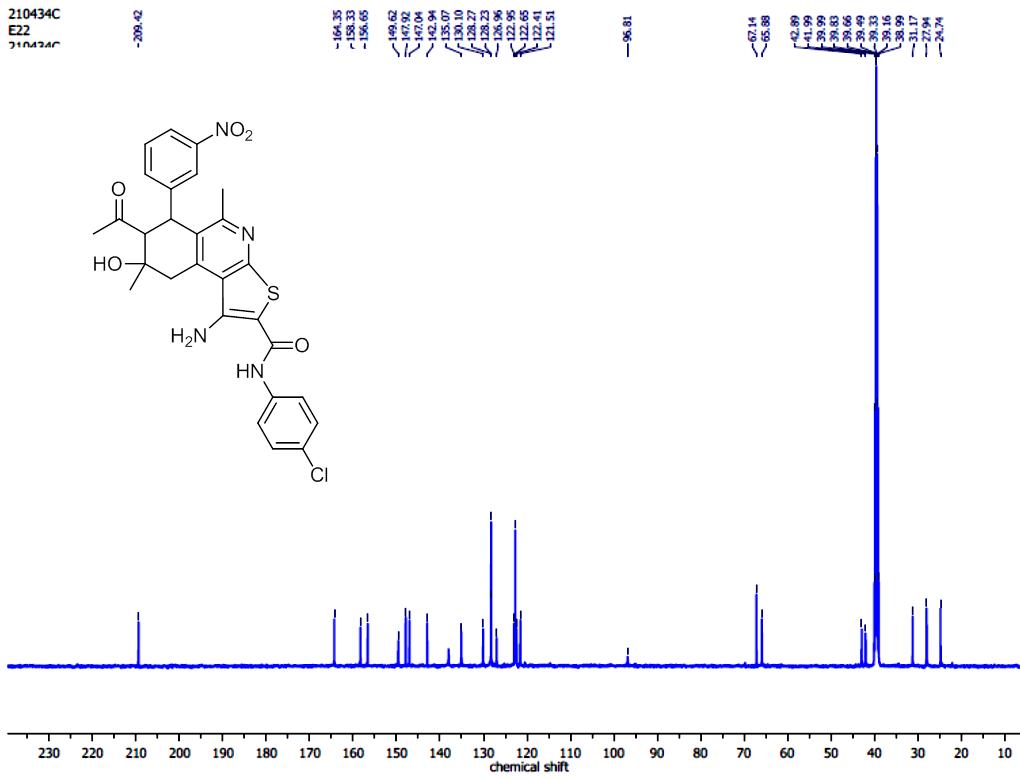
**

**Figure S**30: ^13^ C NMR Spectrum of 7-Acetyl-1-amino-*N*-(4-chlorophenyl)-5,8-dimethyl-8-hydroxy-6-(3-nitrophenyl)-6,7,8,9-tetrahydrothieno[2,3-*c*]isoquinoline-2-carboxamide **(7C)** in DMSO-*d_6_*.

**Figure S31**: FT**-**IR spectrum of the 2-((7-acetyl-4-cyano-6-hydroxy-1,6-dimethyl-8-(3-nitrophenyl)-5,6,7,8-tetrahydroisoquinolin-3-yl)thio)-N-(naphthalen-1-yl)acetamide **(8a)**.


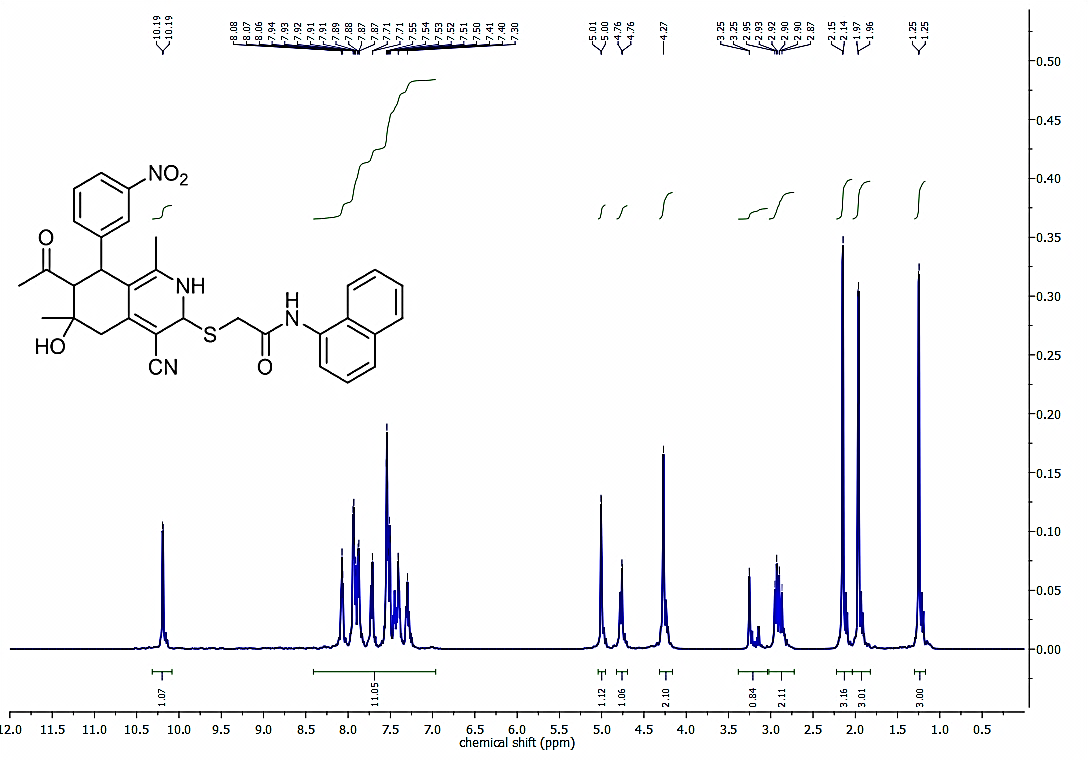


**Figure S32**: ^1^H NMR spectrum of the 2-((7-acetyl-4-cyano-6-hydroxy-1,6-dimethyl-8-(3-nitrophenyl)-5,6,7,8-tetrahydroisoquinolin-3-yl)thio)-N-(naphthalen-1-yl)acetamide **(8a)**.

**Figure S33**: FT**-**IR spectrum of the 2-((7-acetyl-4-cyano-6-hydroxy-1,6-dimethyl-8-(4-nitrophenyl)-5,6,7,8-tetrahydroisoquinolin-3-yl)thio)-N-(naphthalen-1-yl)acetamide  **(8b)**.

**Figure S34**: ^1^H NMR spectrum of the 2-((7-acetyl-4-cyano-6-hydroxy-1,6-dimethyl-8-(4-nitrophenyl)-5,6,7,8-tetrahydroisoquinolin-3-yl)thio)-N-(naphthalen-1-yl)acetamide **(8b)**.

**
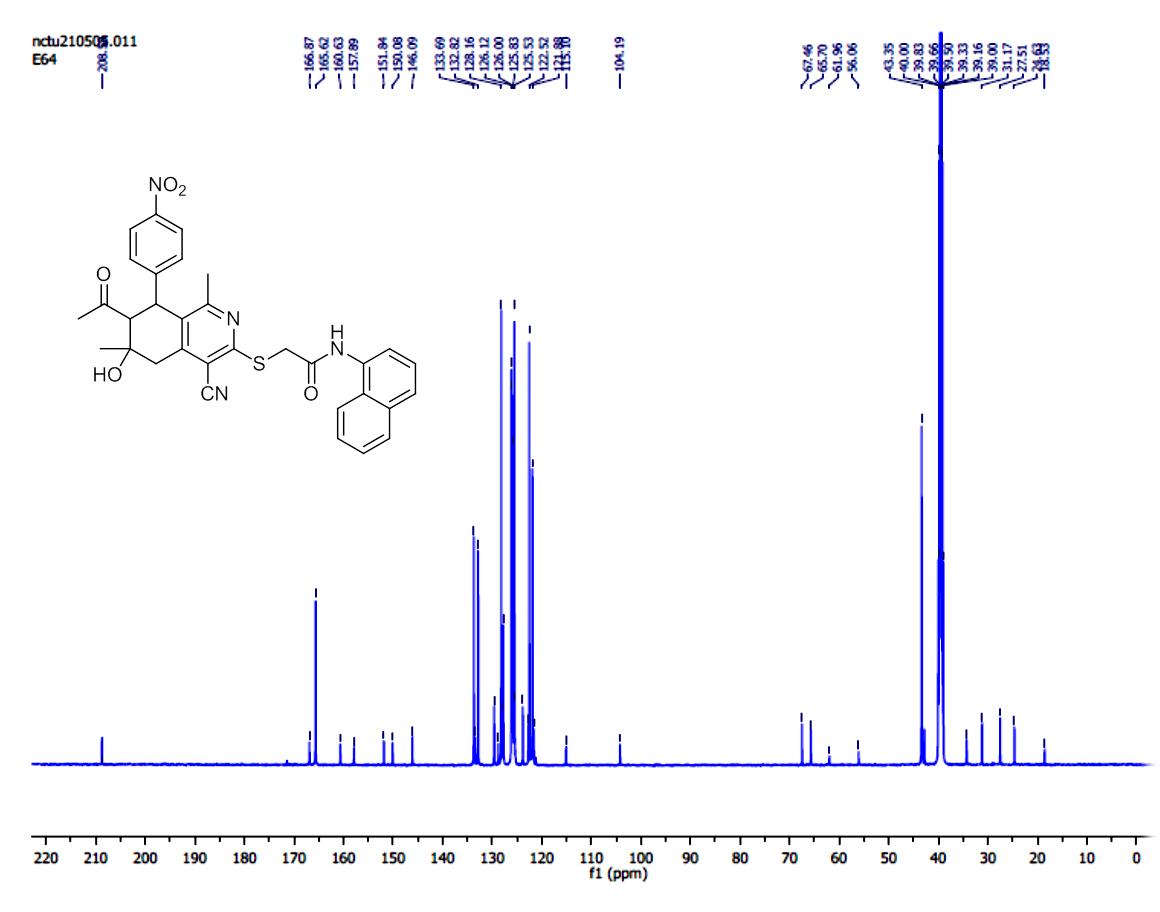
**

**Figure S35**: **^13^C** spectrum of the 2-((7-acetyl-4-cyano-6-hydroxy-1,6-dimethyl-8-(4-nitrophenyl)-5,6,7,8-tetrahydroisoquinolin-3-yl)thio)-N-(naphthalen-1-yl)acetamide **(8b).**

**Figure S** 36: FT-IR Spectrum of 7-Acetyl-1-amino-*N* (naphthalen-1-yl)-5,8-dimethyl-8-hydroxy-6-(3-nitro- phenyl)-6,7,8,9-tetrahydrothieno[2,3-*c*]isoquinoline-2-carboxamide **(9a).**

**
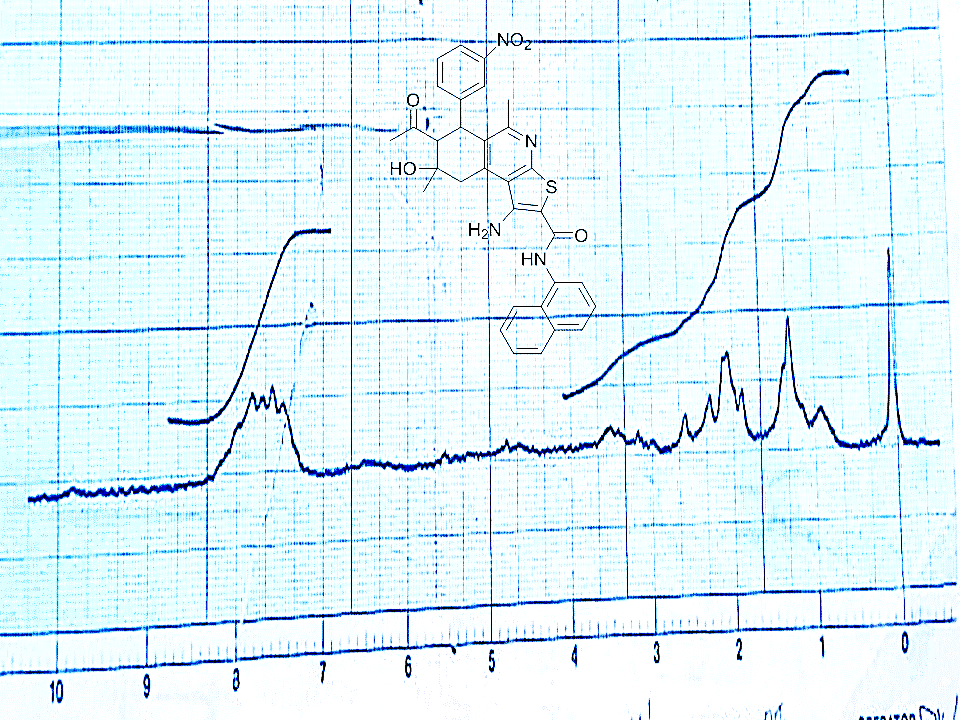
**

**Figure S** 37: ^1^ HNMR Spectrum of 7-Acetyl-1-amino-*N* (naphthalen-1-yl)-5,8-dimethyl-8-hydroxy-6-(3-nitro- phenyl)-6,7,8,9-tetrahydrothieno[2,3-*c*]isoquinoline-2-carboxamide **(9a) in CDCl_3_.**

**Figure S**38: FT-IR Spectrum of 7-Acetyl-1-amino-*N* (naphthalen-1-yl)-5,8-dimethyl-8-hydroxy-6-(4-nitro- phenyl)-6,7,8,9-tetrahydrothieno[2,3-*c*]isoquinoline-2-carboxamide **(9b).**

**
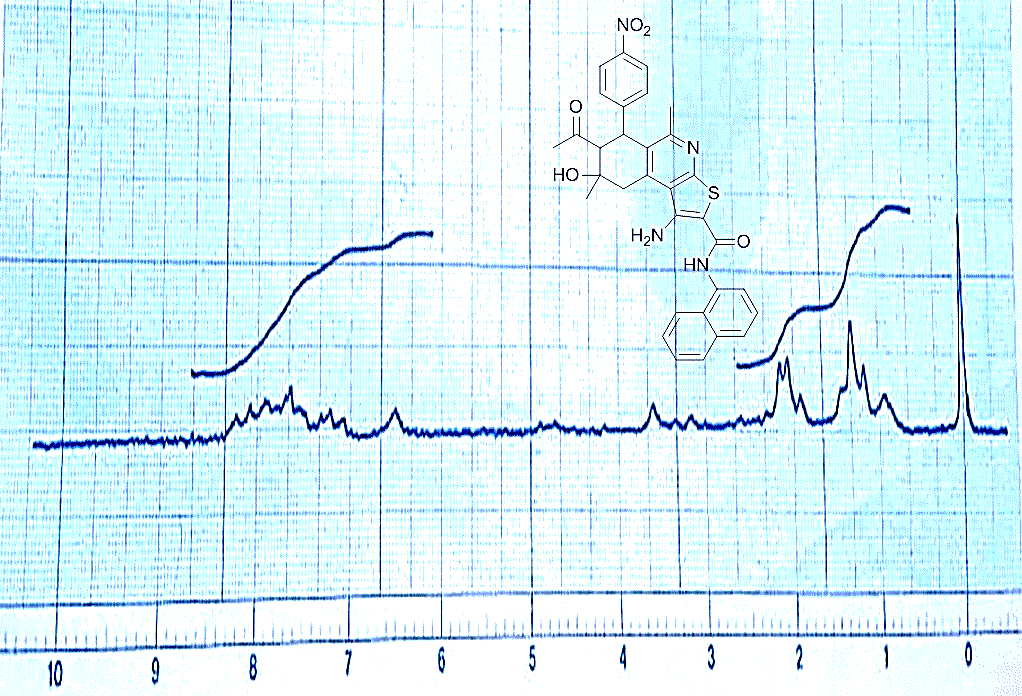
**

**Figure S**39: ^1^H NMR Spectrum of 7-Acetyl-1-amino-*N* (naphthalen-1-yl)-5,8-dimethyl-8-hydroxy-6-(4-nitro- phenyl)-6,7,8,9-tetrahydrothieno[2,3-*c*]isoquinoline-2-carboxamide **(9b)** **in CDCl_3_**_._

**Figure S40**: FT**-**IR spectrum of the 2-((7-acetyl-4-cyano-6-hydroxy-1,6-dimethyl-8-(3-nitrophenyl)-2,3,5,6,7,8-hexahydroisoquinolin-3-yl)thio)-N-phenylacetamide **(10)**.

**
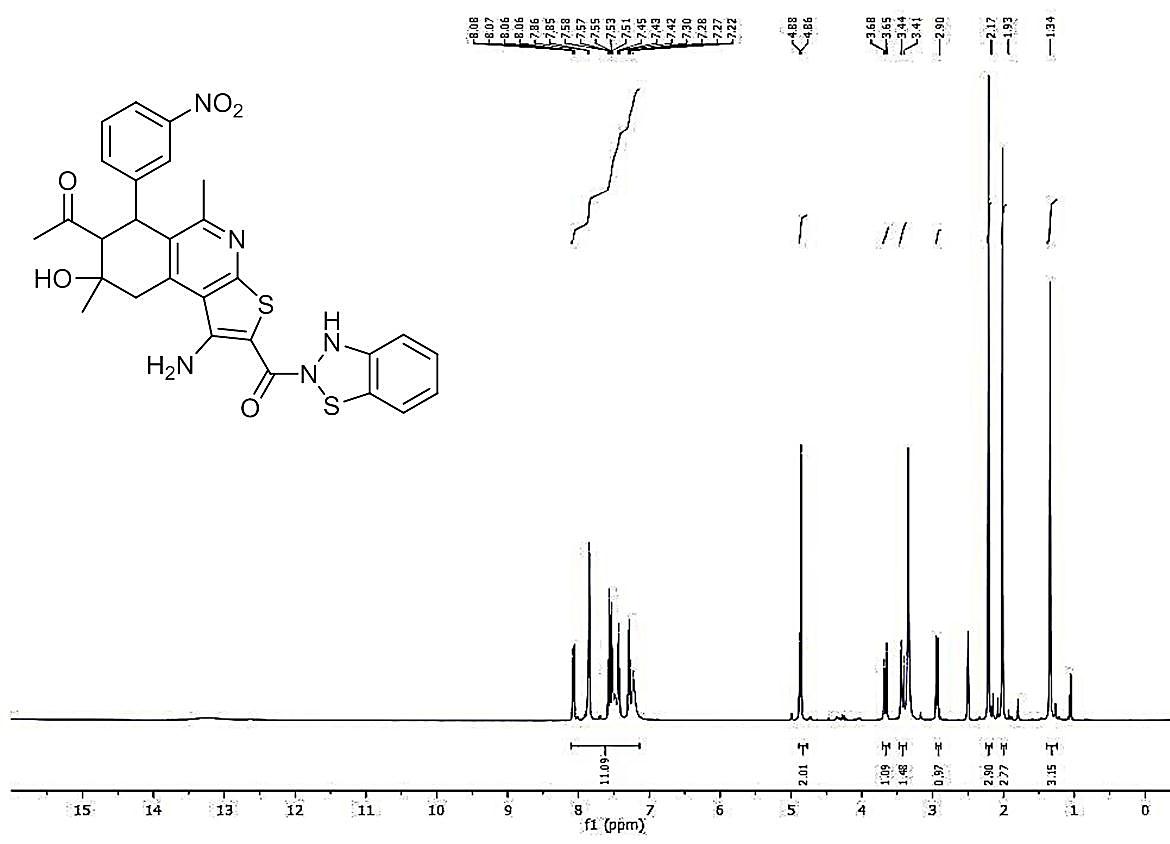
**

**Figure S41**: ^1^H NMR spectrum of the 2-((7-acetyl-4-cyano-6-hydroxy-1,6-dimethyl-8-(3-nitrophenyl)-2,3,5,6,7,8-hexahydroisoquinolin-3-yl)thio)-N-phenylacetamide **(10).**


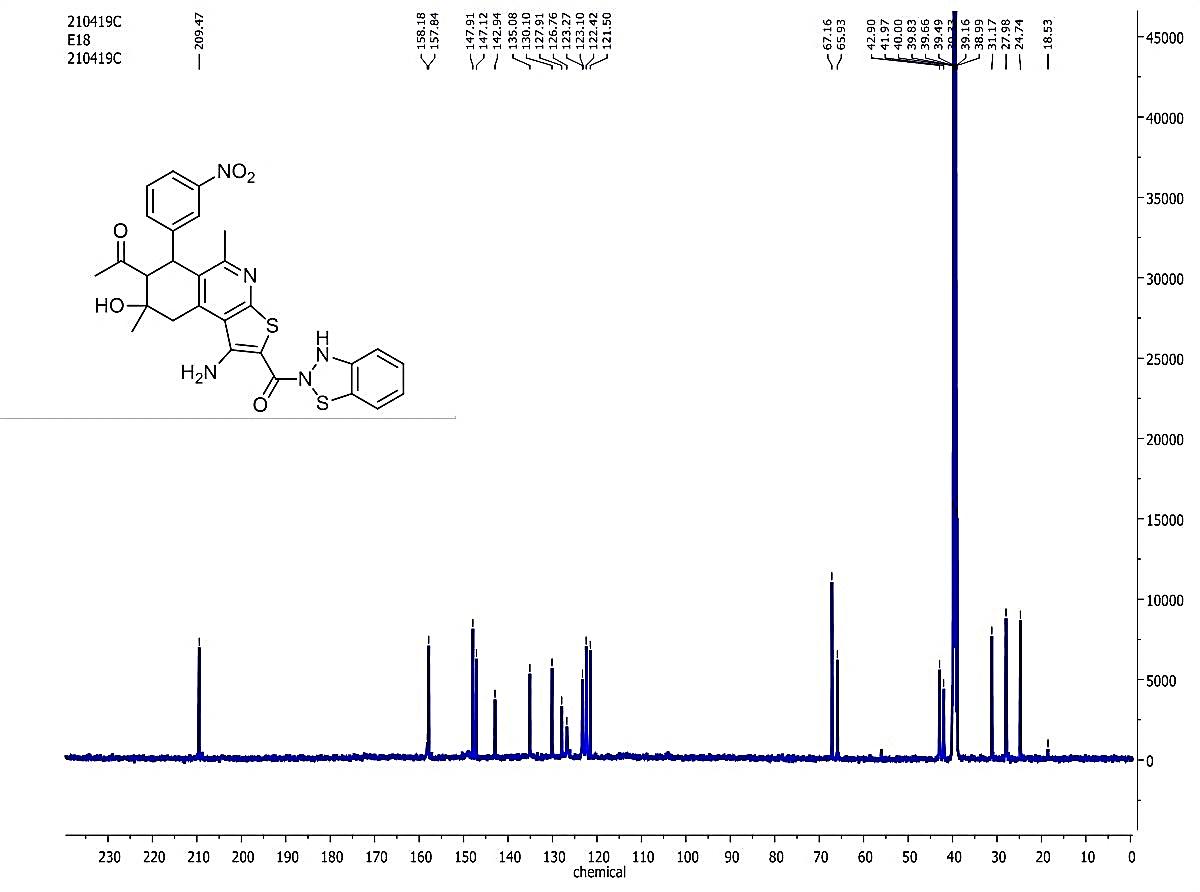


**Figure S42**: **^13^C** spectrum of the 2-((7-acetyl-4-cyano-6-hydroxy-1,6-dimethyl-8-(3-nitrophenyl)-2,3,5,6,7,8-hexahydroisoquinolin-3-yl)thio)-N-phenylacetamide **(10)**.

Table S1: one spot concentration of all synthesized compounds against nine cell lines

| COMPD.NO. | MG-63 | S.D. | HCT116 | S.D. | CACO2 | S.D. | A549 | S.D. |  |  | | |  | |  | |  | |  | |  |
| --- | --- | --- | --- | --- | --- | --- | --- | --- | --- | --- | --- | --- | --- | --- | --- | --- | --- | --- | --- | --- | --- |
| 3 | 44.257467 | 5.0615101 | 67.072323 | 1.5623534 | 59.170306 | 2.9743363 | 50.552251 | 1.2551645 |  |  | | |  | |  | |  | |  | |  |
| 4 | 53.76525 | 1.0201393 | 56.815021 | 2.7360661 | 64.379289 | 1.2773297 | 47.196262 | 2.1565064 |  |  | | |  | |  | |  | |  | |  |
| 5 | 55.742533 | 4.3010107 | 58.936022 | 2.1638884 | 61.540861 | 2.316797 | 55.225149 | 2.5307342 |  |  | | |  | |  | |  | |  | |  |
| 10 | 46.739588 | 2.7157049 | 59.179416 | 4.3827403 | 60.636307 | 4.2070406 | 47.408666 | 2.6924302 |  |  | | |  | |  | |  | |  | |  |
| 2a | 41.901557 | 4.5335982 | 62.308762 | 2.7545622 | 55.1466 | 2.4425168 | 42.438403 | 3.4204367 |  | |  | |  | |  | |  | |  | |  |
| 6a | 45.814051 | 2.9228698 | 58.10153 | 1.2531865 | 54.834685 | 2.7286783 | 20.305862 | 5.7353913 |  | |  | |  | |  | |  | |  | |  |
| 7a | 50.441733 | 2.051956 | 59.700974 | 1.6700709 | 58.203369 | 3.2862301 | 46.813934 | 3.3265543 |  | |  | |  | |  | |  | |  | |  |
| 8a | 38.4939 | 2.012768 | 56.05007 | 3.3139785 | 53.181535 | 0.8893685 | 46.17672 | 0.9899049 |  | |  | |  | |  | |  | |  | |  |
| 8b | 57.509466 | 2.1467986 | 67.524339 | 1.0705705 | 41.671865 | 1.9479095 | 15.208156 | 7.4587528 |  |  | |  | |  | |  | |  | |  | |
| 9b | 30.164072 | 4.0570681 | 55.632823 | 3.4945665 | 51.746725 | 3.607579 | 44.987256 | 1.0987695 |  |  | | |  | |  | |  | |  | |  |
| DOX | 63.819941 | 1.9278821 | 70.862309 | 5.1487372 | 74.828447 | 4.2398668 | 32.030586 | 1.9467186 |  |  | | |  | |  | |  | |  | |  |

Table S1 Continued:

| COMPD.NO. | H460 | S.D. | HUH7 | S.D. | HEPG2 | S.D. | MCF7 | S.D. | HSF | S.D. |
| --- | --- | --- | --- | --- | --- | --- | --- | --- | --- | --- |
| **3** | 62.004326 | 1.6105485 | 32.940847 | 4.2172522 | 67.742789 | 0.6657711 | 71.531792 | 1.7520745 | 50.605971 | 3.7508045 |
| **4** | 66.762797 | 0.903386 | 27.474068 | 3.5025507 | 62.132839 | 2.33886 | 76.32948 | 1.4194253 | 49.423589 | 6.20265 |
| **5** | 66.594569 | 2.5320004 | 35.49201 | 4.2678266 | 68.245568 | 4.2007166 | 67.630058 | 3.0449866 | 66.834171 | 1.365191 |
| **10** | 63.326124 | 1.3398087 | 21.754976 | 3.7142623 | 55.22625 | 1.6404323 | 75.433526 | 0.9024564 | 54.655631 | 1.5994974 |
| **2a** | 55.299207 | 1.9075352 | 27.389964 | 3.9746136 | 61.286055 | 0.4372244 | 73.988439 | 2.6011561 | 46.201596 | 1.3545893 |
| **6a** | 63.47032 | 3.4072211 | 38.631904 | 3.0791054 | 59.486637 | 2.4906658 | 66.358382 | 3.908472 | 53.502808 | 1.8943506 |
| **7a** | 59.865417 | 2.7295882 | 32.352117 | 6.2692024 | 58.666314 | 2.1098385 | 57.919075 | 6.4450219 | 51.847473 | 2.384451 |
| **8a** | 59.552992 | 3.0951821 | 25.876086 | 8.4713243 | 59.962953 | 1.1236239 | 72.369942 | 2.4298092 | 56.902158 | 3.2071785 |
| **8b** | 62.028359 | 2.2026319 | 30.165405 | 5.2187598 | 66.631384 | 3.8116343 | 57.225434 | 2.6488877 | 48.152527 | 5.917157 |
| **9b** | 58.038933 | 4.0886653 | 35.688253 | 1.7847821 | 69.965599 | 1.0719571 | 55.202312 | 1.8049127 | 56.222288 | 1.4197824 |
| **DOX** | 72.891132 | 1.806772 | 52.761424 | 1.2874643 | 58.904472 | 0.5958365 | 73.959538 | 0.1324444 | 58.912208 | 4.1907913 |

Table2: cytotoxicity of the sythnsizedcompounds.

| **compound number** | **Concentration 0** | **Concentration 12.5** | **Concentration 25** | **Concentration 50** | **Concentration 100** |
| --- | --- | --- | --- | --- | --- |
| **3** | 1.0276 | 0.7687 | 0.6657 | 0.63227 | 0.4568 |
|  | 0.9103 | 0.6287 | 0.6563 | 0.62339 | 0.3317 |
|  | 1.0266 | 0.72896 | 0.5889 | 0.61914 | 0.3526 |
| AVERGE | 0.9881667 | 0.7087867 | 0.6369667 | 0.6249333 | 0.3803667 |
| **4** | 0 | 12.5 | 25 | 50 | 100 |
|  | 1.0276 | 0.7104 | 0.7231 | 0.53934 | 0.479 |
|  | 0.9103 | 0.76882 | 0.7277 | 0.49132 | 0.4559 |
|  | 1.0266 | 0.86413 | 0.7515 | 0.53432 | 0.53984 |
| AVERGE | 0.9881667 | 0.7811167 | 0.7341 | 0.52166 | 0.49158 |
| **10** | 0 | 12.5 | 25 | 50 | 100 |
|  | 1.0276 | 0.997437 | 1.0244 | 0.8607 | 0.73312 |
|  | 0.9103 | 0.986 | 0.92 | 0.85914 | 0.64231 |
|  | 1.0266 | 1.1 | 0.9654 | 0.9302 | 0.64832 |
| AVERGE | 0.9881667 | 1.0278123 | 0.9699333 | 0.8833467 | 0.6745833 |
| **2A** | 0 | 12.5 | 25 | 50 | 100 |
|  | 1.0276 | 0.96577 | 0.74688 | 0.64552 | 0.4277 |
|  | 0.9103 | 0.96412 | 0.74415 | 0.64778 | 0.42184 |
|  | 1.0266 | 1.1486 | 0.8787 | 0.7304 | 0.3671 |
| AVERGE | 0.9881667 | 1.0261633 | 0.78991 | 0.6745667 | 0.4055467 |
| **6A** | 0 | 12.5 | 25 | 50 | 100 |
|  | 1.0276 | 0.8094 | 0.7265 | 0.65253 | 0.4895 |
|  | 0.9103 | 0.9004 | 0.7856 | 0.6182 | 0.4568 |
|  | 1.0266 | 0.8971 | 0.8697 | 0.7192 | 0.5797 |
| AVERGE | 0.9881667 | 0.8689667 | 0.7939333 | 0.66331 | 0.5086667 |
| **7A** | 0 | 12.5 | 25 | 50 | 100 |
|  | 1.0276 | 0.9779 | 0.8015 | 0.7673 | 0.4356 |
|  | 0.9103 | 0.9423 | 0.8177 | 0.6711 | 0.5604 |
|  | 1.0266 | 1.0361 | 0.9059994 | 0.7619 | 0.4229 |
| AVERGE | 0.9881667 | 0.9854333 | 0.8417332 | 0.7334333 | 0.4729667 |
|  | 0 | 12.5 | 25 | 50 | 100 |
| **8A** | 1.3043 | 1.1856 | 0.8324 | 0.76209 | 0.5543 |
|  | 1.3043 | 1.1645 | 0.9594 | 0.8064 | 0.6353 |
|  | 1.3043 | 1.0852 | 0.9527 | 0.7671 | 0.6449 |
| AVERGE | 1.3043 | 1.1451 | 0.9148333 | 0.77853 | 0.6115 |
| **8B** | 0 | 12.5 | 25 | 50 | 100 |
|  | 0.9063 | 0.9329 | 0.8067 | 0.68007 | 0.4501 |
|  | 0.8542 | 0.98791 | 0.8494 | 0.636 | 0.3823 |
|  | 0.9211 | 0.8723 | 0.8704 | 0.5876 | 0.417 |
| AVERGE | 0.8938667 | 0.9310367 | 0.8421667 | 0.6345567 | 0.4164667 |
| **9B** | 0 | 12.5 | 25 | 50 | 100 |
|  | 0.9063 | 0.9726 | 0.7934 | 0.6021 | 0.3938 |
|  | 0.8542 | 0.9995728 | 0.7536 | 0.667 | 0.371 |
|  | 0.9211 | 0.899 | 0.7874 | 0.6503 | 0.4041 |
| AVERGE | 0.8938667 | 0.9570576 | 0.7781333 | 0.6398 | 0.3896333 |

Table S3: Cytotoxicity of DOX against HEPG2.

Figure 43: Cytotoxicity of DOX against HEPG2.

Table S4: Cytotoxicity of DOX against MCF7.

Figure 44: Cytotoxicity of DOX against MCF7.

Table S5: interactions of **8b** compound with RET enzyme.

| Interaction | Distance | category | Type |
| --- | --- | --- | --- |
| LYS893 | 2.71327 | Hydrogen Bond | Conventional Hydrogen Bond |
| PRO892 | 3.4556666 | Hydrogen Bond | Carbon Hydrogen Bond |
| VAL892 | 3.75965 | Hydrophobic | Pi-Sigma |
| ARG889 | 5.4818 | Hydrophobic | Pi-Alkyl |
| PRO931 | 4.48016 | Hydrophobic | Pi-Alkyl |

Table S6: interactions of Standard compound with RET enzyme

interactions

| Interaction | Distance | category | Type |
| --- | --- | --- | --- |
| A:GLU935:CA - :UNL1:O | 3.21679 | Hydrogen Bond | Carbon Hydrogen Bond |
| A:ILE890:CD1 - :UNL1 | 3.38069 | Hydrophobic | Pi-Sigma |
| A:HIS903 - :UNL1:C | 4.53641 | Hydrophobic | Pi-Alkyl |

Table S7: interactions of compound **3** with HSP90 enzyme.

| Interaction | distance | Category | Type |
| --- | --- | --- | --- |
| A:TYR305:HH - :UNK1:O | 2.4645 | Hydrogen Bond | Conventional Hydrogen Bond |
| A:ARG378:HE - :UNK1:O | 2.41396 | Hydrogen Bond | Conventional Hydrogen Bond |
| A:ARG378:HH22 - :UNK1:O | 2.71189 | Hydrogen Bond | Conventional Hydrogen Bond |
| A:ASP311:OD2 - :UNK1 | 4.04436 | Electrostatic | Pi-Anion |
| :UNK1:S - A:PHE341 | 5.27227 | Other | Pi-Sulfur |
| A:PHE341 - :UNK1 | 5.2244 | Hydrophobic | Pi-Pi T-shaped |
| A:PHE341 - :UNK1:C | 4.96597 | Hydrophobic | Pi-Alkyl |

Table S8: interactions of standered compound with HSP90 enzyme.

| Interaction | distance | Category | Type |
| --- | --- | --- | --- |
| :UNL1:C - A:ASP364:OD2 | 3.37557 | Hydrogen Bond | Carbon Hydrogen Bond |
| A:ARG378:NH1 - :UNL1 | 4.42611 | Electrostatic | Pi-Cation |
